# The autophagy inhibitor NSC185058 suppresses mTORC1-mediated protein anabolism in cultured skeletal muscle

**DOI:** 10.1038/s41598-024-58716-1

**Published:** 2024-04-06

**Authors:** Patrick J. Ryan, Selina Uranga, Sean T. Stanelle, Megan H. Lewis, Colleen L. O’Reilly, Jessica M. Cardin, J. William Deaver, Aaron B. Morton, James D. Fluckey

**Affiliations:** 1 Muscle Biology Laboratory, Department of Kinesiology and Sport Management, Texas A&M University, Gilchrist Building, 2929 Research Parkway, College Station, TX 77843-4243 USA; 2 Soft Tissue Regeneration and Applied Biomaterials Laboratory, Texas A&M University, Gilchrist Building, 2929 Research Parkway, College Station, TX 77843-4243 USA

**Keywords:** Autophagy, Growth factor signalling, Lysosomes, TOR signalling

## Abstract

The mammalian target of rapamycin (mTOR), and specifically the mTOR complex 1 (mTORC1) is the central regulator of anabolism in skeletal muscle. Among the many functions of this kinase complex is the inhibition of the catabolic process of autophagy; however, less work has been done in investigating the role of autophagy in regulating mTORC1 signaling. Using an in vitro model to better understand the pathways involved, we activated mTORC1 by several different means (growth factors, leucine supplementation, or muscle contraction), alone or with the autophagy inhibitor NSC185058. We found that inhibiting autophagy with NSC185058 suppresses mTORC1 activity, preventing any increase in cellular protein anabolism. These decrements were the direct result of action on the mTORC1 kinase, which we demonstrate, for the first time, cannot function when autophagy is inhibited by NSC185058. Our results indicate that, far from being a matter of unidirectional action, the relationship between mTORC1 and the autophagic cascade is more nuanced, with autophagy serving as an mTORC1 input, and mTORC1 inhibition of autophagy as a form of homeostatic feedback to regulate anabolic signaling. Future studies of cellular metabolism will have to consider this fundamental intertwining of protein anabolism and catabolism, and how it ultimately serves to regulate muscle proteostasis.

## Introduction

**Figure 1:**
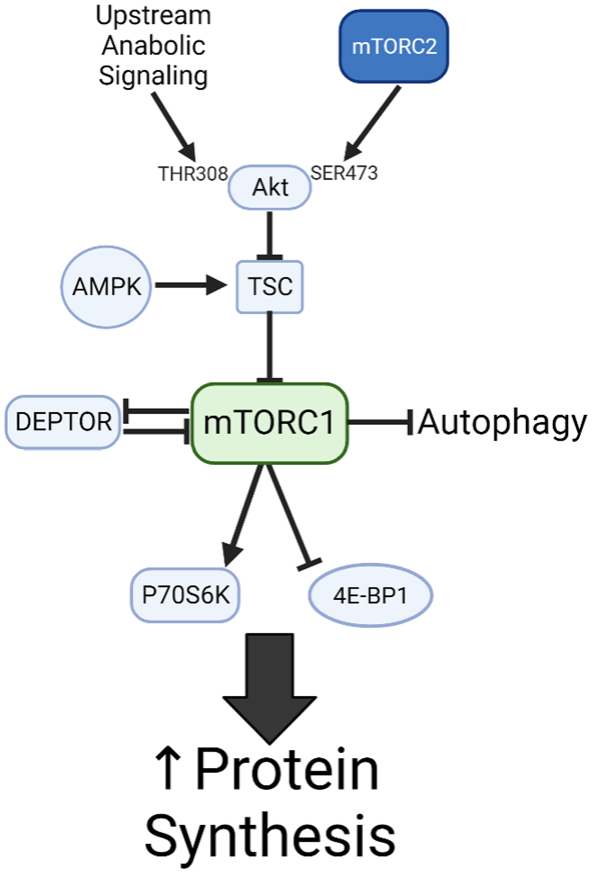
Canonical mTORC1 signaling. The mTOR pathway is a key regulator of cellular anabolic activity, responsive to numerous upstream inputs and capable of altering cellular anabolism by acting on two downstream targets, P70S6K and 4EBP1 to increase protein synthesis, as well as causing numerous other alterations to cellular metabolism (including inhibiting autophagy).

The synthesis of new proteins is fundamental to cellular anabolism. While the basics of protein synthesis, namely the addition of amino acid building blocks to growing polypeptide chains in the ribosome, are simple, the regulation of this process is complex and not completely understood. There are numerous initiation and elongation factors governing the behavior of the ribosome, and several regulatory checkpoints for protein synthesis, but one of the most critical regulators of anabolic behavior is the mechanistic target of rapamycin (mTOR). mTOR is a serine/threonine protein kinase regarded as at least two distinct complexes based on its binding partners, the rapamycin-sensitive mTORC1, primarily promoting protein synthesis and cellular anabolism, and the rapamycin-insensitive mTORC2, likely playing a role in cellular survival^1^. The canonical mTOR pathway is diagramed in Fig. 1 In addition to its role in promoting protein synthesis, mTORC1 promotes mitochondrial biogenesis, lipid metabolism, and is a known negative regulator of the process of autophagy. Indeed, mTORC1-mediated anabolic activity has largely been viewed in opposition to the catabolic process of autophagy, but there is reason to speculate that the relationship between mTORC1 and the autophagic machinery may be more nuanced than previously described. The goal of the present investigation was to reexamine the contributions of autophagy to mTORC1 signaling, further elucidating the interplay between these key anabolic and catabolic pathways.

Macroautophagy (hereafter referred to autophagy) is a highly conserved intracellular process by which proteins and organelles are broken down into their constituent macromolecules. The process of autophagy is also complex, but put simply involves the formation of the autophagosome, a double membrane bound vesicle that encapsulates a target cargo, which fuses with the lysosome, where the contents of the autophagosome are broken down by lysosomal hydrolysis and ultimately recycled. Numerous enzymes (the various members of the ATG family) participate in the autophagic pathway, but an early step in this process is the cleavage of the microtubule-associated proteins 1A/1B light chain 3A (LC3 or ATG8) by the cysteine protease ATG4B^2^. In this way, ATG4B is responsible for catalyzing an initial step of autophagy, and thus represents a key target for understanding the role of autophagic proteolysis to cellular function. The contribution of ATG4B to muscle physiology has not been fully elucidated, but it has been shown that ATG4B is highly expressed in skeletal muscles^3^, and ATG4B-null mice display profound metabolic disruption predominately in muscle^4^, suggesting that this enzyme might play a unique role in regulating muscle metabolism.

Autophagy is critical for cellular health and has largely been studied in the response of the cell to stressful conditions such as nutrient deprivation. This phenomenon has largely been credited to reductions in mTOR activity in response to reduced cellular energetic balance (thought to be mediated by the action of AMPK), with increased autophagy as a result of reduced mTOR activity providing substrate necessary to continue cellular metabolic activity. However, despite the clear role of stimuli promoting mTOR (such as growth factors, exercise, or nutrient stimulation) in negatively regulating autophagy and the demonstrable induction of autophagy with mTOR inhibition, there is growing appreciation that autophagic signaling is in fact critical to cellular growth outside of the response to starvation. For instance, autophagy-deficient mice display profound muscle atrophy^5^, and the knockout of a newly-discovered autophagy gene, MYTHO, in mice leads to pathological changes in muscle, in spite of muscle hypertrophy^6^. Further, evidence suggests that autophagy is required for muscular adaptations to exercise, and that both the mTOR and autophagic pathways are enhanced in response to exercise training^7,8^. Those findings point at a more reciprocal relationship between autophagy and cellular growth than has previously been established.

To investigate the relationship between autophagy and mTORC1 activity in regulating muscle cellular anabolism, we employed a specific inhibitor of ATG4B, NSC185058^9,10^, which acts by fitting into the active site of the ATG4B protease. Muscle is unique in its ability to mount an anabolic response to numerous different stimuli, including growth factors, amino acids, and mechanotransduction from muscle contraction. To tease out the interplay between mTORC1 and autophagy in responding to these inputs, we used cultured rat (L6) myotubes, exposed to supplemental growth factors, leucine, or electrical pulse stimulation, to investigate the effects of each of these anabolic stimulators in the presence or absence of the ATG4B inhibitor NSC185058. Our experiments show, for the first time, that when autophagic flux is inhibited by NSC185058, mTORC1 is unable to respond to anabolic stimulation. These results point to a role for autophagy in anabolic signaling beyond the canonical inhibition in response to anabolism, demonstrating that mTORC1 may be unable to operate without contribution from autophagic proteolysis.

## Results

### NSC185058 suppresses protein anabolism in cultured skeletal muscle

**Figure 2:**
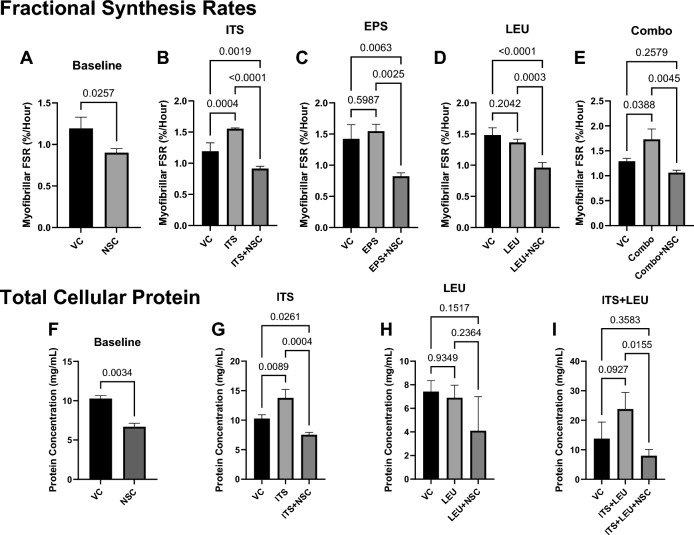
Autophagy is required for protein anabolism in skeletal muscle. Treatment with the ATG4B inhibitor NSC185058 (NSC) reduces myofibrillar protein fractional synthesis rate (FSR, **A**–**E**, n = 3–4) when compared to vehicle control (VC) and prevents the deposition of cellular protein (**F**–**I**, n = 3) both at baseline and in response to a variety of anabolic stimuli (ITS—Insulin, Transferrin, Selenium; LEU—Leucine; EPS—Electrical Pulse Stimulation) either alone or in combination (Combo).

For almost every combination of anabolic stimulators tested, inhibition of autophagy by NSC185058 prevented increases in protein deposition and FSR. NSC185058 administered alone (baseline) caused a 23.8% (*p* < 0.05) reduction in myofibrillar FSR and blunted anabolic behavior in ITS (FSR; VC vs ITS + NSC: − 23.4%, ITS vs ITS + NSC: − 41.2%), EPS (VC vs EPS + NSC: − 42.2%, EPS vs EPS + NSC: − 46.8%, *p* < 0.05), Leu (VC vs Leu + NSC: − 35%, Leu vs Leu + NSC: − 30%, *p* < 0.05) and Combo (VC vs Combo + NSC: − 17.6% *p* > 0.05, Combo vs Combo + NSC: − 38.4%, *p* < 0.05 except where indicated) groups (Fig. 2A–E). These differences in FSR led to similar trends in total accretion of cellular protein, where NSC185058 reduced protein deposition by 34.9% (*p* < 0.05) in the baseline condition. ITS increased protein deposition by 34.1% (*p* < 0.05). No significant differences among conditions were observed in cells treated with leucine alone, while the 72.4% increase observed when ITS was combined with leucine did not rise to the level of statistical significance (IB, *p* > 0.05); however, NSC185058 effectively prevented any of these gains (Fig. 2F–IB, ITS vs ITS + NSC: − 50%, ITS + LEU vs ITS + LEU + NSC: − 66%).

### NSC185058 treatment blunts mTORC1 activity

**Figure 3:**
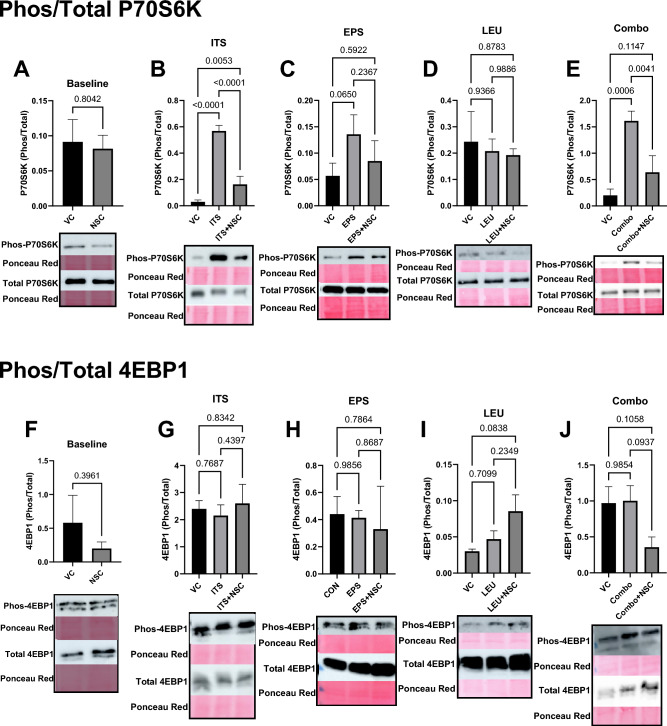
Autophagic Inhibition Blunts mTORC1-Mediated Anabolic Signaling. Inhibition of ATG4B by NSC185058 (NSC) prevents mTORC1 phosphorylation of P70S6K (**A**–**E**, n = 3–4) in in response to a variety of anabolic stimuli (ITS—Insulin, Transferrin, Selenium; LEU—Leucine; EPS—Electrical Pulse Stimulation) either alone or in combination (Combo), with no difference when compared to vehicle control alone (VC). We found no increase in phosphorylation of 4EBP1 (**F**–**J**, n = 3–4) in any condition. Representative cropped blot images are displayed below bar graphs, with original uncropped images available in Supplementary Fig. S3.

We next set out to determine the mechanism underlying the demonstrated reduction in anabolic protein metabolism with autophagic inhibition by analyzing the phosphorylation status of the downstream effectors of the growth-promoting mTORC1 kinase, P70S6K and 4EBP1. While there were no differences in the Baseline condition, similar to our measurements of protein synthesis, the ratio of phosphorylated to total P70S6K was elevated in the ITS (VC vs ITS: 1800%, *p* < 0.05) and Combo conditions (VC vs Combo: 700%, *p* < 0.05), with no differences observed with EPS (despite a trend towards a 140% increase, *p* = 0.065) or LEU treatment (Fig. 3A–E). NSC185058 treatment blunted the increase in P70S6K phosphorylation in every condition where it was stimulated (VC vs ITS + NSC: 450%, ITS vs ITS + NSC: − 71%, VC vs Combo + NSC: 220%, Combo vs Combo + NSC: − 40%, *p* < 0.05), with no difference between the EPS + NSC and LEU + NSC groups relative to their respective vehicle controls (Fig. 3A–E). We observed no differences in the phosphorylation of 4EBP1 in any condition (Fig. 3F–J), which we speculate might relate to a temporal difference as to when these targets are activated during the anabolic signaling cascade. As supplemental leucine exposure had no effect on protein deposition, FSR, or P70S6K phosphorylation, likely because the cell media already contained sufficient leucine to stimulate mTOR activity, this group was removed from further analysis. In contrast to the observed effect on mTORC1, NSC185058 treatment had no effect on phosphorylation of the anabolic ERK pathway (Supplemental Fig. S1), indicating that the effects of autophagy inhibition on anabolic behavior are distinct to the mTOR axis.

**Figure 4:**
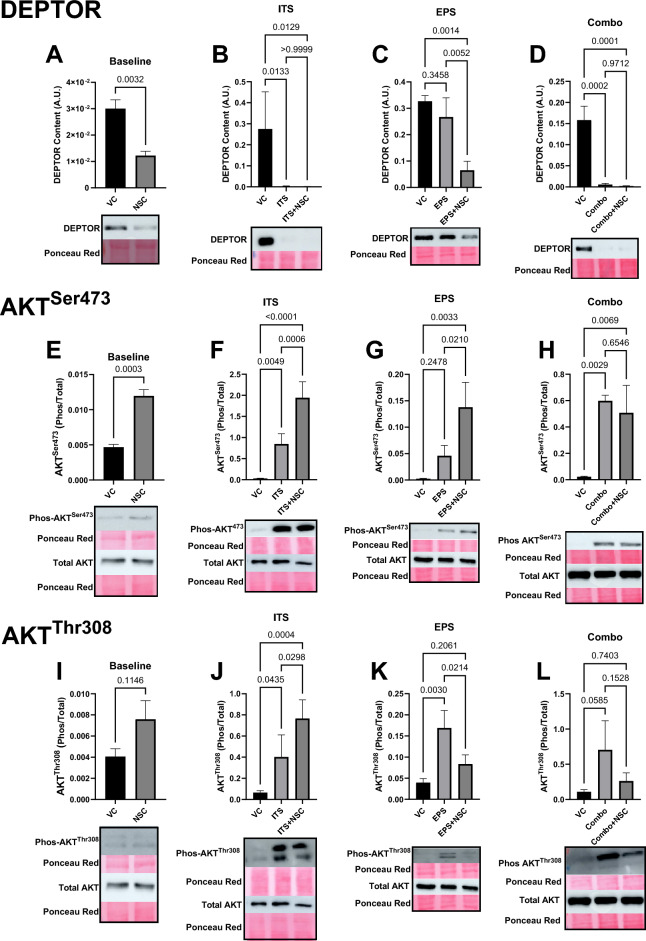
NSC185058 Does Not Alter Upstream mTORC1 Effectors. Treatment with NSC185058 (NSC) did not affect reductions in DEPTOR (**A**–**D**, n = 3–4) content, AKT^Ser473^ phosphorylation (**E**–**H**, = 3–4), or AKT^Thr308^ phosphorylation (**I**–**L**, n = 3–4), with anabolic stimulation (ITS—Insulin, Transferrin, Selenium; LEU—Leucine; EPS—Electrical Pulse Stimulation; Combo—combination), indicating that the effects of autophagic inhibition are specific to the mTORC1 kinase and cannot be attributed to alterations in upstream signaling of mTORC1, or the endogenous mTOR inhibitor, DEPTOR. In fact, DEPTOR content was lower and AKT^Ser473^ phosphorylation was increased when comparing NSC185058 to vehicle control in the Baseline, ITS, and EPS conditions. Cropped representative images of blots are below each graph, with full blot images available in Supplementary Fig. S4.

We then assessed the content of the endogenous mTOR inhibitor DEPTOR. DEPTOR protein content was reduced in ITS and Combo treatment condition (VC vs ITS: − 99%, VC vs Combo: − 96%, all *p* < 0.05), an effect that was unaffected by NSC185058 (VC vs ITS + NSC: − 99%, VC vs Combo + NSC: 98.2%, *p* < 0.05), which reduced DEPTOR levels by 59% (*p* < 0.05) in the Baseline condition and 83% in EPS (*p* < 0.05) (Fig. 4A–D). These results demonstrate that the observed reductions of mTORC1 activity are not attributable to alterations in DEPTOR, and in fact show that there is no activity of the mTORC1 kinase despite dramatic reductions in DEPTOR content.

### Autophagy inhibition by NSC185058 does not alter upstream AKT/mTOR pathway signaling

We next set out to determine why mTORC1 activity was being altered by autophagic inhibition, initially investigating the upstream effector of the mTORC1 kinase, AKT. Anabolic stimulation by ITS (VC vs ITS: 2900%, *p* < 0.05) and Combo (VC vs Combo: 2500%, *p* < 0.05), though not EPS (VC vs EPS: 1400%, *p* > 0.05), significantly increased AKT^Ser473^ phosphorylation, with no observed detriment due to NSC185058 (F, VC vs ITS + NSC: 3800%, VC vs EPS + NSC: 5500%, VC vs Combo + NSC: 2000%, *p* < 0.05)—in fact phosphorylation of AKT^Ser473^ was also increased by 156% with NSC185058 treatment alone at baseline (Fig. 4E–H). AKT^Thr308^ phosphorylation was elevated in ITS (VC vs ITS: 760%, *p* < 0.05) and EPS (Fig. 4C, VC vs EPS: 430%, *p* < 0.05), but not Combo (*p* > 0.05) and unaffected at Baseline and ITS with NSC185058 (Fig. 4I–L), indicating that the effects of NSC185058 treatment on mTORC1 activity are not attributable to any influence on the chief upstream effector of the mTOR pathway, AKT. Further, colocalization analysis of mTOR and LAMP2 indicates that mTOR kinase translocation to the lysosomal membrane is promoted by Combo treatment and unaffected by NSC185058 (Supplemental Fig. S2, VC vs Combo: 18%, VC vs Combo + NSC: 16%, *p* < 0.05) demonstrating that the normal machinery that allows for mTORC1 activity in response to anabolic stimuli is unaltered by autophagic inhibition.

### Amino acid availability and cellular energetic balance do not mediate the effect of NSC185058

**Figure 5:**
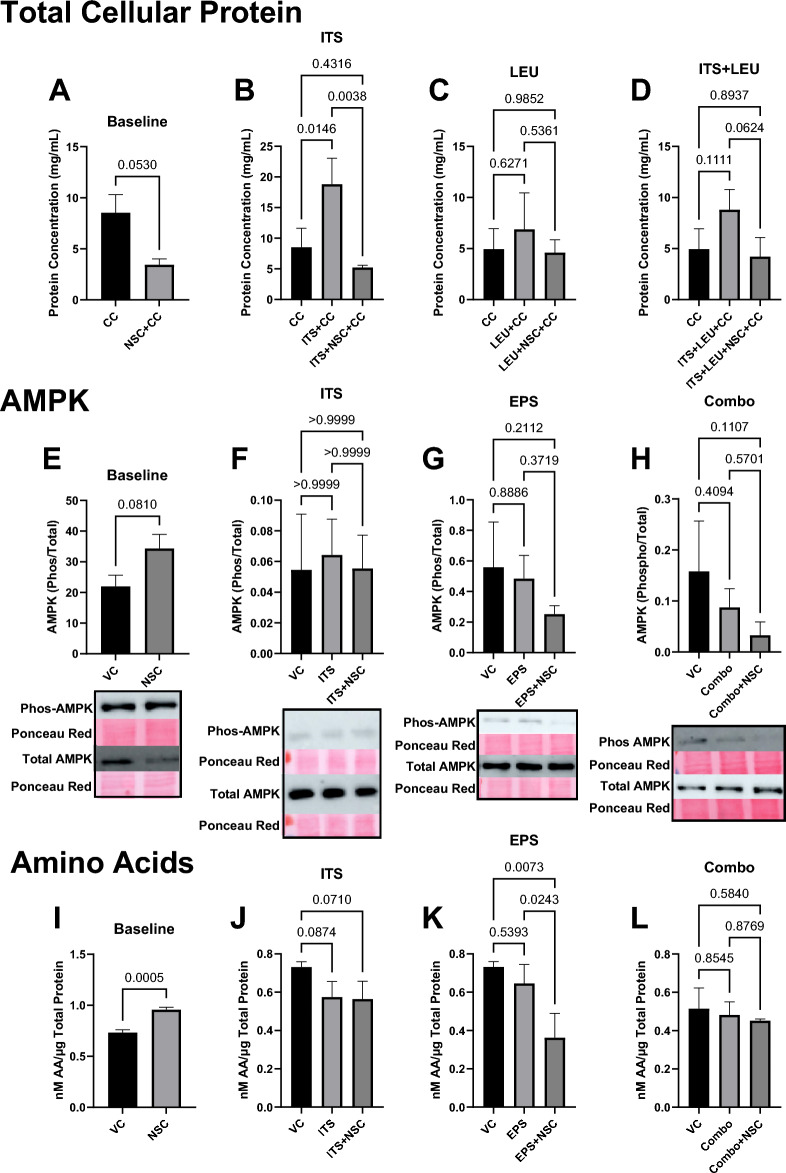
Inhibition of Autophagy Does Not AMPK Activity or Amino Acids in Skeletal Muscle. Treatment with the AMPK inhibitor Compound C (Dosomoprhin) does not mediate the reductions in total protein deposition induced by NSC185058 (NSC), further indicating that the effect of autophagy inhibition is independent of AMPK activity (**A**–**D**, n = 3). Further, administration of NSC185058 (NSC) does not impact AMPK phosphorylation (**E**–**H**, n = 3–4), or normalized cytosolic amino acid concentration (**I**–**L**, n = 3) indicating that the effects of NSC185058 are independent of these factors (VC—Vehicle Control; ITS—Insulin, Transferrin, Selenium; LEU—Leucine; EPS—Electrical Pulse Stimulation; Combo—combination). Full Western blot images are available in Supplementary Fig. S5.

One possible explanation for these observations is that the autophagic pathway contributes to either intracellular energetic balance or amino acid levels to such a degree that inhibition of this process would lead to subsequent effects on cellular anabolism. To test the first hypothesis, we performed Western immunoblots for the phosphorylation status of AMPK, a cellular energy sensor and known inhibitor of mTORC1, and repeated our protein deposition assay with the addition of Compound C (also known as dorsomorphin), an AMPK inhibitor. Patterns of protein deposition were largely recreated despite the addition of Compound C (Fig. 5A–D), and no significant difference in AMPK phosphorylation was observed except in the Combo condition, where it was significantly lower (*p* < 0.05) than the vehicle control value (Fig. 5E–HB). To test the second hypothesis, we assayed cytosolic cell lysate fractions for total amino acid content. NSC185058 treatment did lead to reduced amino acid levels in the EPS condition (*p* < 0.05); however, it also *increased* amino acid content at baseline (*p* < 0.05) with no change in the ITS or Combo groups, which are notably the conditions where FSR was increased (Fig. 5I–L), making bulk changes in substrate availability an unlikely contributor for the observed outcomes. These results indicate that the effect of NSC185058 on mTORC1-mediated anabolism is likely not due to alterations in upstream signaling, substrate availability, or cellular energetic balance.

### Autophagic flux is required for mTORC1-mediated anabolism in skeletal muscle

**Figure 6:**
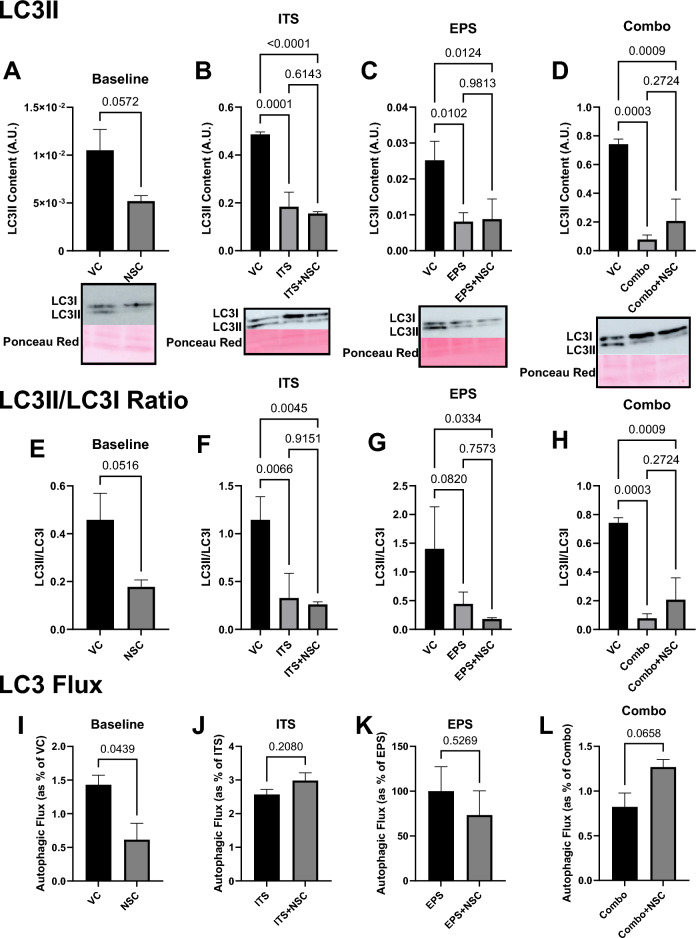
Autophagic Flux is Required for mTORC1-Mediated Anabolism in Skeletal Muscle. Anabolic stimulation reduced LC3II protein content (**A**, n = 3), LC3II/I ratio (**B**, n = 3), and autophagic flux (**C**, n = 3), as would be expected from the documented inhibitory effect of mTORC1 on the autophagic signaling cascade. No differences were observed between groups receiving stimulators (ITS—Insulin, Transferrin, Selenium; LEU—Leucine; EPS—Electrical Pulse Stimulation; Combo—combination), alone or with co-administered NSC185058 (NSC). However, NSC185058 reduced autophagic flux when administered alone versus vehicle control (VC), indicating that the action of the lysosome is in some way required for mTORC1 to respond to anabolic stimulation. Cropped representative images are shown below graphs in (**A**); full blot images are available in the Supplementary Fig. S6.

mTORC1 has been demonstrated to suppress autophagic activity. Accordingly, Western immunoblots of LC3B demonstrate a reduction in both LC3II content (Fig. 6A–D) and LC3II/1 ratio (Fig. 6E–H) with anabolic stimulation that activates mTORC1. Similar effects are observed with NSC185058 treatment, with no difference in either LC3II/I ratio or total LC3II content between cells with active mTORC1 and those with mTORC1 activity inhibited by NSC185058 (Fig. 6A–H). A plausible mechanistic explanation for these findings emerges when considering the measurement of autophagic flux. As both anabolic stimulation and drug treatment inhibited autophagy in these experiments, autophagic flux was not different between muscle cells receiving anabolic stimulation or anabolic stimulation plus NSC185058. However, NSC185058 does lead to a 60% reduction (*p* < 0.05) in flux when administered alone (Fig. 6I–L). We thus propose a novel explanation for these results: namely that flux through the autophagic pathway is required for mTORC1 activity and resultant cellular anabolism, and that when this process is inhibited by NSC185058, there can be no increase in mTORC1-mediated anabolic behavior.

## Discussion

**Figure 7:**
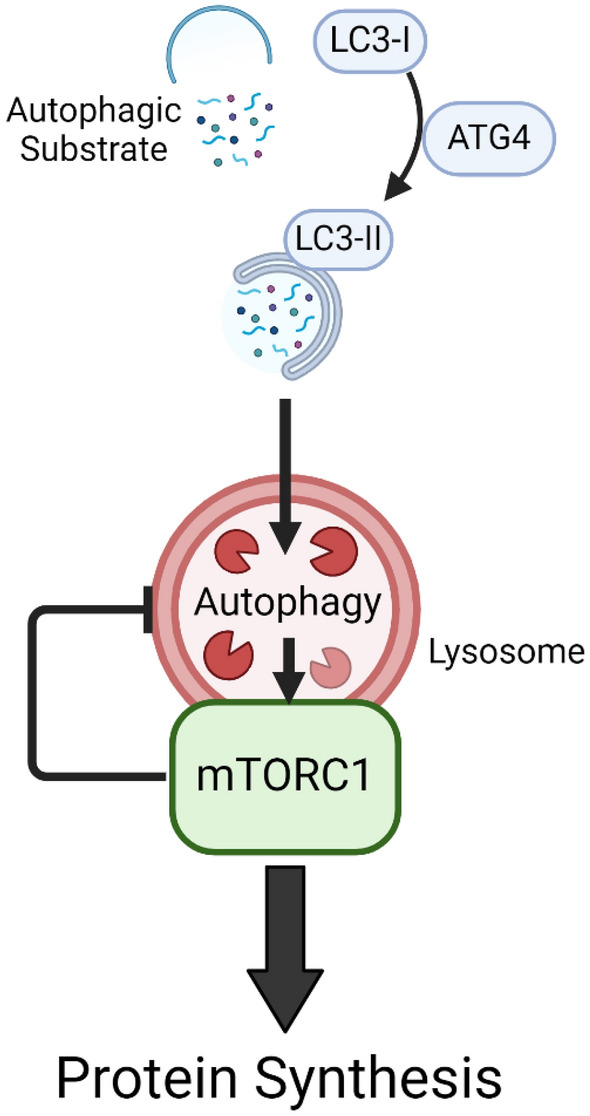
An Updated View of mTORC1 Signaling. Based on our results, autophagy plays a role as an mTORC1 input, and is required for increases in cellular anabolism. Far from a unidirectional action, this view posits that there is a complex and dynamic relationship between mTORC1 and autophagy, such that autophagy is required for increases in mTORC1 activity while mTORC1 inhibits the autophagic cascade, and that the interplay between these processes is a key homeostatic mechanism regulating cellular anabolism in health and disease.

Our main finding is that the autophagy inhibitor NSC185058 suppresses mTORC1-mediated protein synthesis, the accretion of cellular protein and anabolic signaling in skeletal muscle. While mTORC1 has long been shown to inhibit autophagy, these results are the first to indicate that the reverse is also true: namely, that autophagic proteolysis is required for the mTORC1-mediated response to anabolic stimuli. The chief implication of these results is that the autophagic signaling cascade, while largely studied in relation to reduced cellular energy state and cell stress, is also a key player in the adaptation of muscle to conditions which stimulate growth as well. As can be seen in our experiments, cultured muscle that possesses all the requisite cellular machinery and substrate to mount an anabolic response cannot do so in the presence of autophagic inhibition by NSC185058. This hints at a more nuanced understanding of cellular protein metabolism, indicating that rather than being viewed as separate metabolic pathways, protein anabolism and catabolism are inextricably linked, and that the synthesis of new proteins depends in large part upon contributions from the autophagic pathway. In this way, the canonical understanding of protein synthesis (Fig. 1) may require an update, with the mTOR-mediated inhibition of autophagy being more fully viewed as a form of negative feedback to help regulate anabolic signaling and thereby cellular homeostasis (Fig. 7).

Several observations made in the course of this investigation support this proposed mechanism. NSC185058 administration in L6 myotubes reduced both total protein deposition and myofibrillar protein FSR compared to vehicle control treated cells. While we detected no change in P70S6K or 4EBP1 phosphorylation in this baseline conditions, subsequent experiments demonstrated that NSC185058 blunted increases in protein deposition, FSR, and P70S6k phosphorylation in response to every single and combined anabolic stimulus utilized. These findings are specific to the mTOR pathway, as markers of the distinct ERK signaling cascade were not influenced by autophagic inhibition. Further, the nature of the interplay between autophagy and mTOR-mediated protein synthesis is not related to disruptions in cellular energetic status, as we found no clear pattern of AMPK activation with NSC185058 treatment, and the AMPK inhibitor compound C did not alter protein deposition patterns with anabolic stimulation. Changes in bulk substrate availability for protein synthesis are also an unlikely explanation, as total cytosolic amino acid content was similarly not uniformly altered by NSC185058, and was especially unaltered by drug administration in conditions that stimulated increases in FSR. In addition,, it seems that there is no defect in canonical signaling upstream of the mTOR kinase, as AKT phosphorylation was still present, DEPTOR content was still reduced, and mTOR translocation still occurred during autophagy inhibition with anabolic stimulation. Indeed, it is remarkable that there was no anabolic response in NSC185058 treated cells, given that DEPTOR, an endogenous mTOR inhibitor, content was so low. Finally, while we cannot completely exclude the possibility of off-target effects of NSC185058, we were able to find no alterations in any known canonical regulator of mTORC1 activity; in fact, it is clear that NSC185058 does alter the serine/threonine kinase activity of the central mTOR molecule, as mTORC2 target (AKT^SER473^) phosphorylation was not suppressed by drug administration.

After eliminating altered upstream signaling, perturbed energetic balance, and reduced total substrate availability as explanations for our observations, the most likely remaining explanation for our findings is that there is a direct mechanistic link between autophagy and protein anabolism. This model represents an expansion of the current dogma, which treats mTORC1 as a unidirectional autophagy inhibitor. Indeed, in our experiments, autophagic measures were lower with anabolic stimulation in both the presence and absence of NSC185058—with the key distinction that autophagic flux was effectively inhibited by the drug at baseline. A clearer picture then begins to emerge when considering that both mTORC1-mediated protein synthesis and autophagic proteolysis both share a common hub in the lysosome. When autophagic flux, which terminates in the lysosome, is inhibited, mTORC1 (which translocates to the lysosome) activity is also inhibited, even though all the requisite conditions for its unimpeded function are present. Thus, we propose that the only remaining explanation for our results is that flux through the autophagic pathway is directly required for mTORC1-mediated anabolism, and that autophagy is a necessary input for mTORC1 activity. These findings support and add depth to a growing body of evidence that shows that the lysosome serves as a crucial signaling hub of the cell^11,12^, and indicate that the lysosome, instead of a means of protein disposal, should more properly be viewed as an organelle of cellular adaptation, required for cellular responses to both the stresses of energy deprivation and cellular growth. Our results demonstrate that lysosomal degradation of substrate supplied by the autophagic machinery of the cell is essential to protein synthesis. While this effect is unlikely to be due to changes in free intracellular amino acid levels or detriments in energetic balance, we are currently unable to definitively say what precise contribution autophagy is making to the anabolic process. One logical explanation is that autophagic activity is required for structural remodeling of the cell, given the well-documented role of autophagy in responding to other cellular stresses—perhaps autophagy is needed to respond to any perturbance in cellular homeostasis. Indeed, prior investigations have found that autophagy is required for the remodeling in bone^13,14^, myocardium^15,16^, and skeletal muscle^17–19^. It is worth noting that many of those studies consider autophagy as a survival process that contributes to cell survival in response to a pathological stimulus, be it myocardial infarction or muscle regeneration; whereas, our data suggest that the autophagic cascade is required for more than just cell survival. It certainly seems plausible that the cellular remodeling through the autophagic pathway is required for the addition of protein in response to an anabolic stimulus as well.

However, our results do not preclude the possibility that trafficking of amino acids specifically through the lysosome is required for protein synthesis. Previous studies have shown that recycling of amino acids through autophagy is required to sustain protein synthesis during starvation^20,21^, but we believe our results to be the first to indicate that the contribution of flux through the autophagic pathway is equally essential for the anabolic response of the cell. This idea is not by itself entirely novel; indeed, independent investigations have long documented that recycling of amino acids in tissues outside of muscle is likely required for protein synthesis^22–24^. One recent study suggested^25^ that this contribution is unlikely to come from the ubiquitin proteosome system, finding that inhibition of the proteosome led to subsequent decrements in protein synthesis in C2C12 muscle cells with no contribution to recycling from proteasomal degradation. While we did not observe a change in cytosolic amino acid concentration, we cannot preclude the possibility that a specific trafficking of amino acids through the lysosome is ultimately required for mTORC1 activation. Further experiments will be needed to determine if autophagic proteolysis serves as a means of specific amino acid recycling for protein synthesis, as a more general route for cellular remodeling, or potentially both.

It should be noted that our findings stand somewhat in contrast to those of Akin and colleagues, who found that while NSC185058 treatment slowed growth in osteosarcoma, it did not appear to alter PI3K or mTOR activity^9^. Our results are partially in agreement with those, as NSC185058 treatment in our experiments did not alter phosphorylation of AKT^THR308^, a target of PI3K. In contrast, the present investigation clearly diverges when considering the effect of NSC185058 on mTOR signaling. It is possible that this divergence arises in the differences of cell type under consideration, as the lab group of Akin were investigating the role of autophagy inhibition by NSC185058 in osteosarcoma, while we used differentiated skeletal muscle. However, separate studies of NSC185058 in osteosarcoma demonstrate that drug treatment suppressed osteoclastogenesis^10^, a process that is reliant on mTORC1 activity^26^, giving some additional credence to our conclusion that NSC185058 acts to suppress mTORC1 by inhibiting autophagy. Few studies have probed the relationship between ATG4B, NSC185058 and mTOR, and we believe ours to be the first to do so in skeletal muscle specifically. However, some corroboration with our conclusions comes from the cancer literature, where genetic ablation of ATG4B or NSC185058 treatment has been show to slow cell growth in glioblastoma, while ATG4B knockout resulted in impaired mTORC1 activity in several cancer cell lines. Clearly more work in identifying the finer points of the interaction between ATG4 and mTORC1 activity remains to be done, including more precise genetic manipulations in skeletal muscle. Despite this, we posit that our results provide clear preliminary evidence that NSC185058 slows mTORC1-mediated anabolism by inhibiting autophagy, potentially a novel requisite input for muscle anabolic activity.

The direct immediate applications of these findings may be narrow, but the implications for further investigation into the role of anabolism in health and disease are broad. Prior investigations have shown that autophagy support muscular adaptations to exercise training^27^, while other basic science studies support the notion that autophagy is required for the maintenance of muscle mass^5,6^. Those findings, along with our proposed mechanism, support the concept that autophagy and anabolism are inherently intertwined, and suggest that healthy growth and adaptation in skeletal muscle are likely driven by a complex interplay between the mTOR and autophagic pathways to promote anabolic adaptations. The role of autophagy in muscle atrophy more broadly is less clear. Dysregulation of the autophagic pathway has negative consequences for muscle mass and metabolism in pathologies such as diabetes, where rescue of autophagic flux has been demonstrated to reduce skeletal muscle atrophy^28^, and in conditions such as sepsis, where a recent paper found that muscle-specific ablation of autophagy worsens outcomes, including muscle wasting^29^. Sarcopenia with aging has been associated with impaired autophagy, including a concomitant reduction in protein synthesis in one study^30^, and those findings certainly jibe with our proposed model, indicating that impaired autophagy with aging may underlie decrements in anabolism in sarcopenic muscle. However, the role of the mTOR pathway in regulating muscle mass during atrophy is not entirely clear, with some studies documenting reductions in the activity of mTORC1 and reductions in protein anabolism^31^, while others show a paradoxical elevation of activity of the kinase and protein synthesis during sarcopenia^32^. On the other hand, in muscle wasting from cancer cachexia, various studies have found that autophagic degradation of protein is elevated in models of cachectic pathology^33,34^, with others showing that mTORC1 activity is repressed^35^. Upon first glance this may appear divergent with our model, but there is evidence to suggest that the behavior of autophagy in cachexia may be more complex. Specifically, it appears that while autophagic flux may be induced during cachexia, there is likely a blockage of autophagosome-lysosome fusion^36^, with one study demonstrating that regular exercise (a known activator of mTORC1) is protective against muscle wasting and mortality in cachectic mice^37^, potentially by relieving this load on the lysosome. Taken together, those results support the notion of an interplay between autophagy and mTORC1-regulated anabolism, and suggest that disruptions in the homeostasis between the autophagic and mTOR pathways are crucial for the development and viable targets for the treatment of muscle-wasting disease.

Much more work remains in identifying the implications and physiological relevance of our proposed mechanism. The role of autophagy in cell growth is still controversial. Studies in drosophila show that depletion of ATG9 leads to increased TOR activity^38,39^, although this relationship is somewhat confounded as ATG9 also plays a role in mobilizing lipid droplets to the mitochondria as well as the autophagosome^40^. It is worth noting that one study has found that NSC185058 administration in high fat diet-fed mice had no effect on the response of mice to either moderate or high-intensity aerobic exercise^41^; however, the response of animals to resistance based training (a known stimulator of muscle growth and mTORC1), or training with a normal diet, in combination with ATG4B inhibition are as yet unknown. In contrast, exercise adaptations in ATG6 knockout mice are suppressed relative to wild-type controls^42^, supporting the idea that there is a concrete link between autophagy and adaptations to anabolic stimuli. Although we believe our proposed model to be a valuable improvement on how the relationship between mTORC1 and the autophagic cascade are viewed, there are undeniably many more aspects and implications that remain to be fully identified. For instance, the recent identification of the MYTHO protein as being required for muscle autophagy also demonstrates that MYTHO deletion leads to aberrant muscle growth and mTORC1 activation^6^, but also induces severe myopathy. This phenotype is reminiscent of myostatin deficiency, which leads to an increase in muscle mass but reductions in specific force and contractility^43^, and it may be salient to note that myostatin is a documented inducer of autophagy, including that of the ATG4B gene^44^. Future experiments could also focus on the effects of autophagy enhancement on the mTOR pathway, potentially by using mTOR-independent inducers of autophagy such as lithium or trehalose, or by genetic overexpression of ATG4B. While there is currently no consensus as to how anabolic and catabolic protein pathways affect each other, we believe that our results join others in showing that these processes are inextricably linked, and that a full understanding of muscle anabolism, critical to the study of human health and disease, will have to consider this bidirectional intertwining of muscle proteostasis.

## Methods

### Cell culture and reagents

L6 myoblasts were obtained from ATCC (ATCC Cat# CRL-1458, RRID:CVCL_0385, Manassas, VA, USA) and cultured in Dulbecco’s modified essential media (DMEM; 11995040; Gibco, Grand Island, NY, USA) with 10% fetal bovine serum (1500-500; Avantor, Radnor, PA, USA), and 1% penicillin/streptomycin (Avantor, Radnor, PA, USA) and maintained in a humidified incubator at 37 °C with an atmosphere of 5% CO_2_. All cell culture dishes and plates were obtained from Avantor (45000-652, Avantor, Radnor, PA, USA). For fractional synthesis rate, Western immunoblotting, and amino acid content experiments, myoblasts were grown to a density of approximately 80% in 6-well plates, then switched to sodium pyruvate-free DMEM containing 2% horse serum (Avantor, Radnor, PA, USA) and 1% penicillin/streptomycin to induce differentiation to myotubes, with fiber morphology confirmed by microscope examination after 5 days. For total protein deposition, myoblasts were seeded in a 96-well plate at a density of 10,000 cells/well, then switched to low-serum sodium pyruvate-free DMEM the following day and differentiated for five days.

### Autophagic inhibition and anabolic stimulation

The ATG4B inhibitor NSC185058 (a generous gift of the laboratory group of William A. Dunn, who developed the compound) at a dosage of 100 μM was used for autophagic inhibition. For anabolic stimulation, insulin-transferrin-selenium (ITS, 25-800-CR, Corning Incorporated, Corning, NY, USA), L-leucine (LEU, J62824.36, ThermoFisher Scientific), or electrical pulse stimulation (EPS, achieved with a purpose-built stimulator using graphite electrodes connected to a Grass stimulator set to deliver bipolar pulses of 30 V at 100 Hz for 200 ms every 5th second for 1 h) were used either alone or in combination (Combo) with each other to promote anabolic activity and signaling in cultured muscle cells. Separate groups of cells received these stimulators simultaneously with NSC185058 to investigate the influence of autophagy inhibition on protein synthesis and cellular anabolic signaling, with vehicle control cells receiving an isomolar dose of DMSO. All groups were treated with the indicated combination of stimulator and inhibitor for 24 h, in media further supplemented with 4% deuterium oxide as a stable isotopic tracer for protein synthesis measurements (in the case of the EPS and Combo groups this 24-h started immediately after the end of contractions). Following the 24-h treatment period, cells were washed twice in ice-cold phosphate-buffered saline, then harvested in ice-cold lysis buffer using cell scrapers. Lysates were gently agitated for 1 h with intermittent vortexing before being stored at -80˚ C for further analysis.

### Protein deposition

To determine the effect of autophagy inhibition on total protein deposition, L6 myoblasts were differentiated into myotubes 96-well plates for five days, then treated with the indicated stimulator and inhibitor concentrations in triplicate for an additional 48 h. As the previously described cell-stimulator was constructed to work with 6-well plates, this method was not used to measure protein content in the EPS or Combo groups, but was used to determine total protein in baseline, ITS, LEU, and an ITS + LEU conditions. In order to minimize variation arising from cell harvesting, myotubes were lysed in the wells of the plate, and constantly agitated on a shaker for 1 h. After cell lysis, a BCA assay (23227, ThermoFisher Scientific, Waltham, MA, USA) was conducted to determine the total protein concentration of each well according to manufacturer’s instructions.

### Protein synthesis measurements

Fractional synthesis rates (FSR) were measured using deuterium oxide incorporation as described in^45^. Briefly, cell lysates were centrifuged at 14,000 RPM for 30 min, with the supernatants, containing cytosolic signaling proteins, aspirated and dedicated for Western blot and amino acid content analysis. The remaining pellet, containing myofibrillar proteins, was resuspended in 10% trichloroacetic acid (TCA), then centrifuged again at 3800 RPM for 15 min to separate proteins from amino acids. This process was repeated for a total of three TCA washes, and the final resulting pellet was hydrolyzed in 6N HCL overnight in a heating block set to 100 °C. After hydrolysis to amino acids, samples were derivatized using a 3:2:1 ratio solution of methyl-8, acetone, and acetonitrile, then loaded onto a gas-chromatograph-mass spectrometer, and the enrichment of cellular alanine (E_A_) was determined. Separately, cell media was thawed then incubated in a solution of 10 N NaOH in 5% (vol/vol) acetone:acetonitrile for 24 h, then mixed with N-hexane and extracted for analysis. Enrichment of the media (E_CM_) was calculated using gas chromatography-mass spectrometry (GCMS, Agilent 7890a GC/5975c VL MSD, Santa Clara, CA, USA), and protein fractional synthesis rate was calculated using the equation $\frac{{E}_{A}}{{E}_{CM}\times 3.7\times t\left(h\right) }\times 100$*,* where E_A_ represents amount of protein-bound [^2^H]alanine (mole% excess), E_CM_ is the quantity of ^2^H_2_O in cell media (mole% excess), 3.7 represents the exchange of ^2^H between cell media and alanine (e.g., 3.7 of 4 carbon-bound hydrogen of alanine exchange with water), and *t*(*h*) is the duration of tracer exposure measured in hours.

### Western immunoblotting

Cell lysates containing cytoplasmic proteins were obtained as described above. Cytosolic protein concentration was calculated using a BCA assay, and samples were mixed with 4x Laemmli buffer before being resolved on a polyacrylamide gel for 20 min at 1000 V, 40 mA and 100 W, followed by 60 min at 1000 V, 80 mA and 100 W. All target proteins were run on 12% polyacrylamide gels, with the exception of LC3, which was run on a 4–20% gradient gel (XPO4205, ThermoFisher, Waltham, MA, USA) for clearer separation of the LC3I from LC3II bands. Following SDS-PAGE, samples were transferred to a fortified nitrocellulose membrane (10120-018, VWR, Radnor, PA, USA) for 55 min at 1000 V, 250 mA, and 4 W. Equal loading was verified by Ponceau S staining, and membranes were subsequently blocked in 5% milk for 1 h, washed three times in TBS, then incubated in primary antibody solution at a concentration of 1:1000 on a rocker in a 4 °C refrigerator overnight. Primary antibodies used were: Phosphorylated P70S6^Thr389^ Kinase (Cat# 9205 (also 9205L, 9205S), RRID:AB_330944), Total P70S6 Kinase (Cat# 9202 (also 9202L, 9202S), RRID:AB_331676), Phosphorylated 4EBP1^Thr37/46^ (Cat# 2855 (also 2855L, 2855S, 2855P), RRID:AB_560835), Total 4EBP1 (Cat# 9644 (also 9644S, 9644P), RRID:AB_2097841), Phosphorylated AMPKα^Thr172^ (Cat# 50081, RRID:AB_2799368), Total AMPKα (Cat# 2532 (also 2532L, 2532S), RRID:AB_330331), Phosphorylated AKT^Thr308^ (Cat# 9275, RRID:AB_329828), Phosphorylated AKT^Ser473^ (Cat# 9271 (also 9271S, 9271L, NYUIHC-310), RRID:AB_329825), Total AKT (Cat# 4691 (also 4691L, 4691P, 4691S), RRID:AB_915783), Phosphorylated ERK^Thr202/Tyr204^ (Cat# 4370 (also 4370L, 4370S, 4370P, 4370T), RRID:AB_2315112), Total ERK (Cat# 4695 (also 4695P, 4695S), RRID:AB_390779), and LC3A/B (Cell Signaling Technology Cat# 4108 (also 4108S), RRID:AB_2137703), all from Cell Signaling Technologies (Danvers, MA, USA). After this period, membranes were washed three times in TBS, incubated in goat anti-rabbit conjugated HRP secondary antibody (Cell Signaling Technology Cat# 7074 (also 7074S, 7074V, 7074P2), RRID:AB_2099233) of concentrations from 1:5000 to 1:10,0000 solution for1 h, washed again three times in TBS, and finally incubated in SuperSignal West Pico PLUS chemiluminescent substrate (34578, ThermoFisher, Waltham, MA, USA) for one minute. Detection of the indicated probes was achieved using a Cytvia ImageQuant 800 biomolecular imager, and densitometric analysis was conducted using Cytvia ImageQuant400 software, with signal intensities of measured bands normalized to the intensity of whole-lane Ponceau red staining.

### Autophagic flux

For autophagic flux measurements, 40 μM chloroquine according to standard methods^46,47^ was added to separate groups of cells receiving either anabolic stimulators alone or in combination with NSC185058. All groups of cells were treated for 24 h, then washed twice in ice-cold PBS, removed from the plate in similarly ice-cold lysis buffer with a cell scrapper, and gently agitated for 1 h with intermediate vortexing, before being stored at − 80 °C for Western blot analysis using an anti-LC3A/B antibody (specifics above).

### Amino acid content

Amino acid content was determined by enzymatic colorimetric detection using an L-amino acid quantitation kit (MAK002, Sigma, St. Louis, MO, USA). Briefly, aliquots of cytosolic cell lysate were diluted 1:1 with assay buffer, along with amino acid standards of known quantity. Samples and standards were then dispensed in clear 96-well plates, followed by the addition of an enzyme mix, and incubated at 37 °C in the dark before being read on a spectrophotometer at 570 nm. Sample values were obtained by interpolation to the standard curve and normalized to the total amount of cytosolic protein as calculated by BCA assay.

### Immunostaining and confocal microscopy

L6 myoblasts were grown and differentiated on #1 thickness glass coverslips coated in poly-l-lysine (GG-25-PLL, Neuvitro Corporation, Vancouver, WA, USA) for five days, then subjected to Combo treatment to promote mTORC1 activity. Following treatment, the slides were washed twice in ice-cold PBS, then fixed in ice-cold acetone for 30 min, washed in PBS, and blocked in 1% BSA with 22.25 mg/mL glycine in PBS for 1 h. Coverslips were washed in PBS, then incubated in primary antibody solution containing rabbit anti-mTOR and mouse anti-LAMP1, washed in PBS, then incubated in secondary solution containing goat anti-rabbit antibody conjugated to AlexaFluor 594 (Cat# A-11012, RRID:AB_2534079), ThermoFisher, Waltham, MA, USA) and goat anti-mouse antibody conjugated to AlexaFluor 488 (Cat# A-11001 (also A11001, A 11,001), RRID:AB_2534069), ThermoFisher, Waltham, MA, USA) to visualize mTOR localization to the lysosome. Finally, coverslips were washed in PBS, and incubated in 10 mg/mL wheat germ agglutin (WGA) conjugated to AlexaFluor 555 (W32464, ThermoFisher, Waltham, MA, USA), then mounted in ProLong Gold Antifade Mountant (P10144. ThermoFisher, Waltham, MA, USA) and affixed to microscope slides. Fluorescent staining was visualized and images were acquired with a 63x oil immersion objective (NA = 1.2) with a 2x optical zoom on a Leica Stellaris 5 White Light Laser Confocal Microscope coupled to a camera integrated with LasX software for Lightning deconvolution (Leica Biosystems, Deer Park, IL, USA). Multiple myofibers were randomly sampled across each treatment condition, with slides incubated in secondary antibody solution without primary antibody serving as controls. The Coloc2 tool in ImageJ^48^ (RRID:SCR_003070) was used to first apply thresholds to the images, then obtain Spearman’s correlation coefficients for the 594 and 488 channels to determine colocalization of mTOR and LAMP1.

### Statistical analysis

Differences in the Baseline (VC vs NSC) were determined by a two-tailed t-test, while differences in all other groups (ITS, LEU, EPS, and COMBO) were determined using a one-way ANOVA with Tukey’s post-hoc test in the case of a significant difference. Differences below are presented as percent change. All α levels were set at 0.05. GraphPad Prism (GraphPad Software, Boston, MA, USA) software was used for analysis.

### Supplementary Information

Supplementary Figures.

## Data Availability

Data will be made available upon request to the corresponding author.
