# Supplementary material for: The autophagy inhibitor NSC185058 suppresses mTORC1-mediated protein anabolism in cultured skeletal muscle
Source: Sci Rep. 2024 Apr 6;14:8094. doi: 10.1038/s41598-024-58716-1 (PMC10998866; doi:10.1038/s41598-024-58716-1)
Supplement: Supplementary file 1 — Supplementary Figures. [file 41598_2024_58716_MOESM1_ESM.pdf]

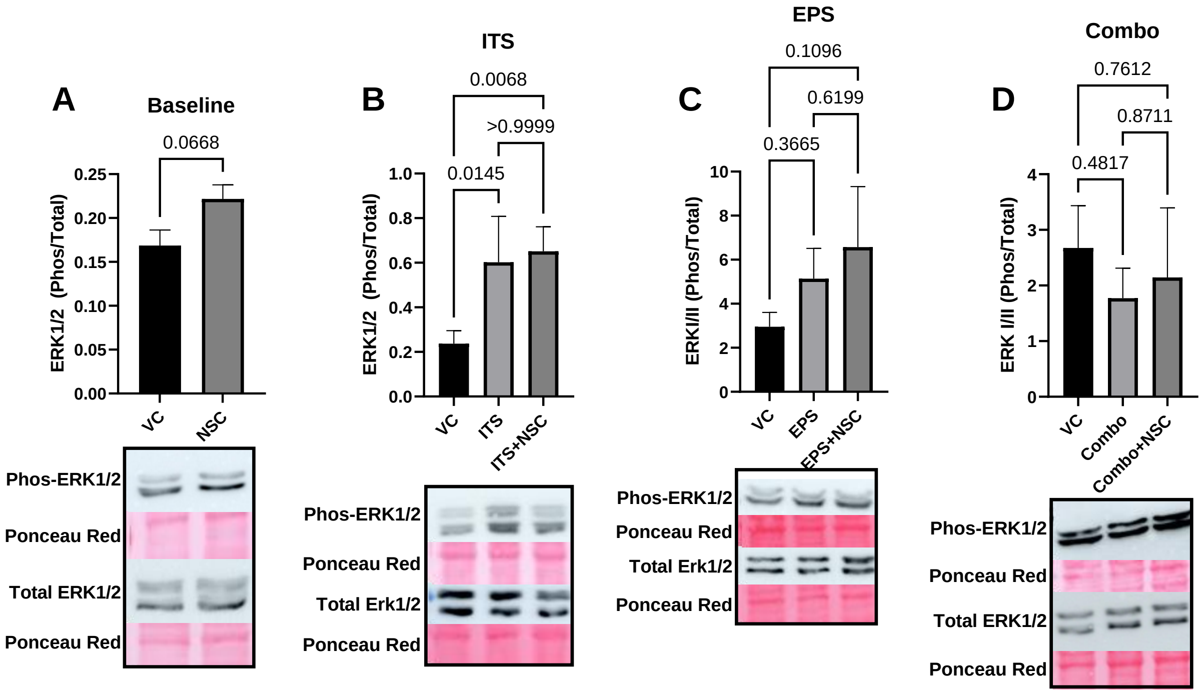

Supplemental Fig. S1: The Effects of NSC185058 on Anabolic Signaling are Specific to the mTOR Pathway. NSC185058 (NSC) treatment did not alter ERK phosphorylation in any condition (Panel A, VC – Vehicle Control; Panel B, ITS – Insulin, Transferrin, Selenium; Panel C, EPS – Electrical Pulse Stimulation; Panel D, Combo – combination), (n=3-4). Full Western blot images are provided below

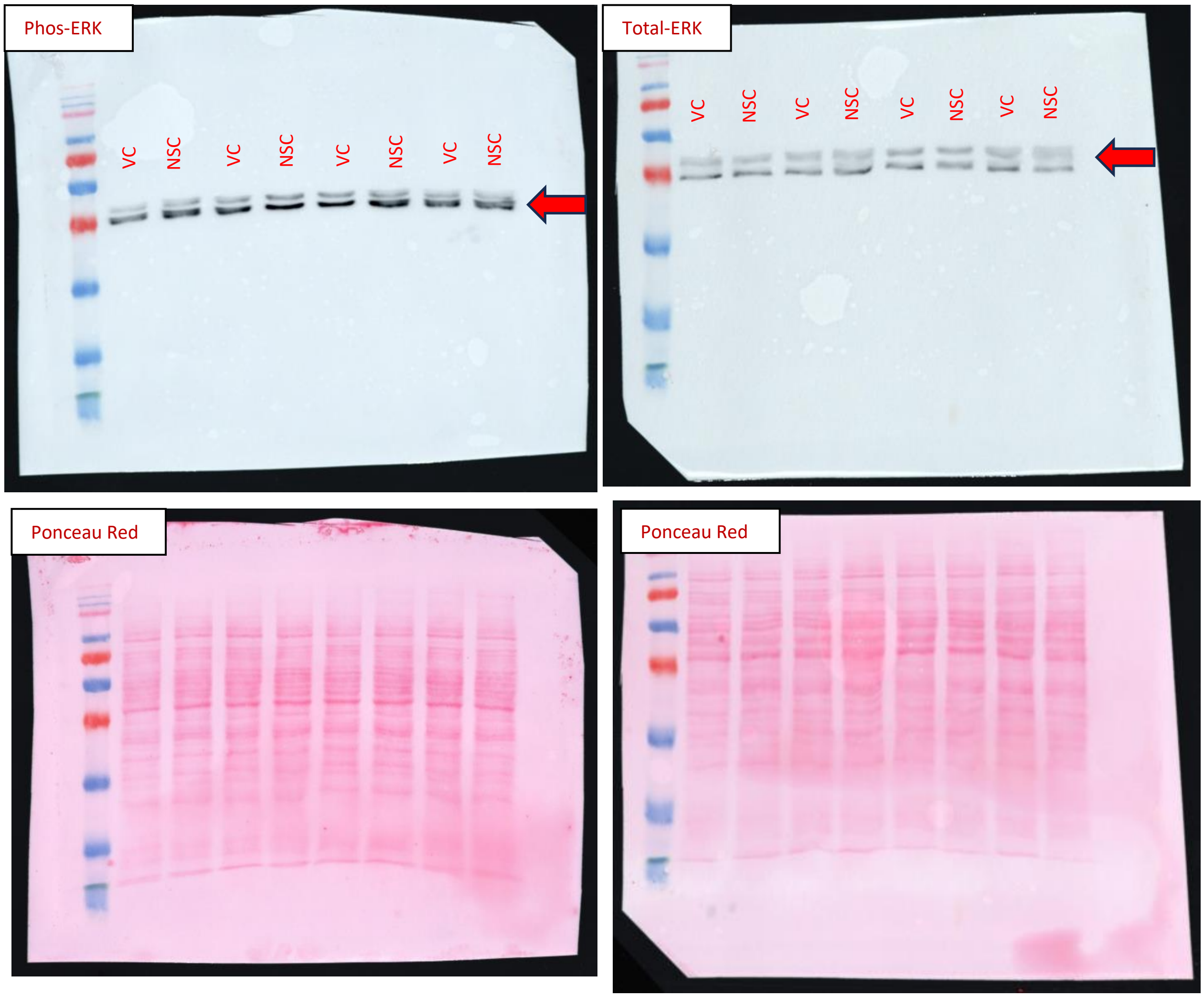

Supplemental Figure S1.A: Full images of phosphorylated ERK (left) and total ERK (Right) in the Baseline condition, without (VC) or with (NSC) NSC185058 treatment. Full Ponceau S stains are presented below each image.

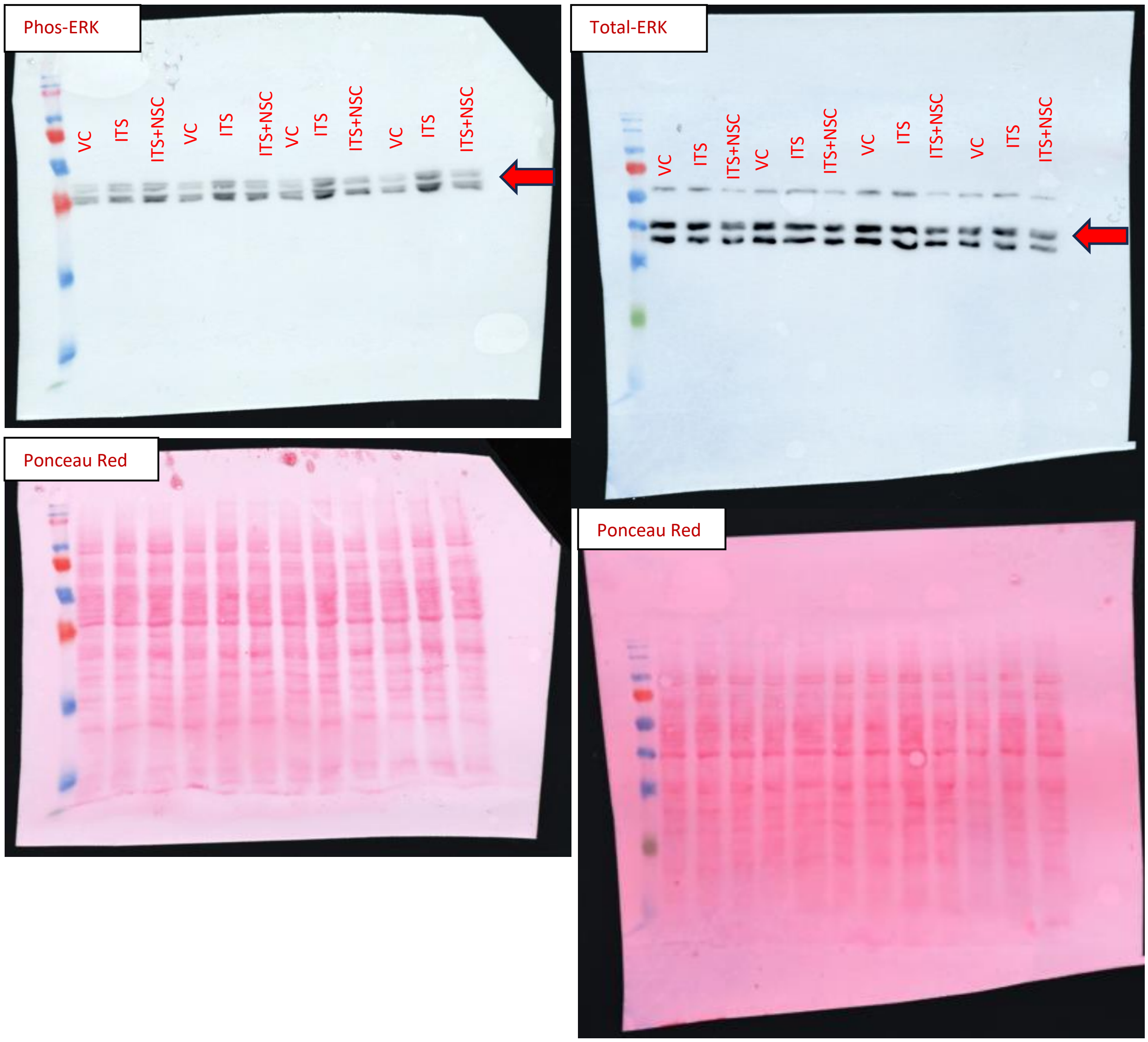

Supplemental Figure S1.B: Full images of phosphorylated ERK (left) and total ERK (Right) in the ITS condition, without (ITS) or with (ITS+NSC) NSC185058 treatment. Full Ponceau S stains are presented below each image.

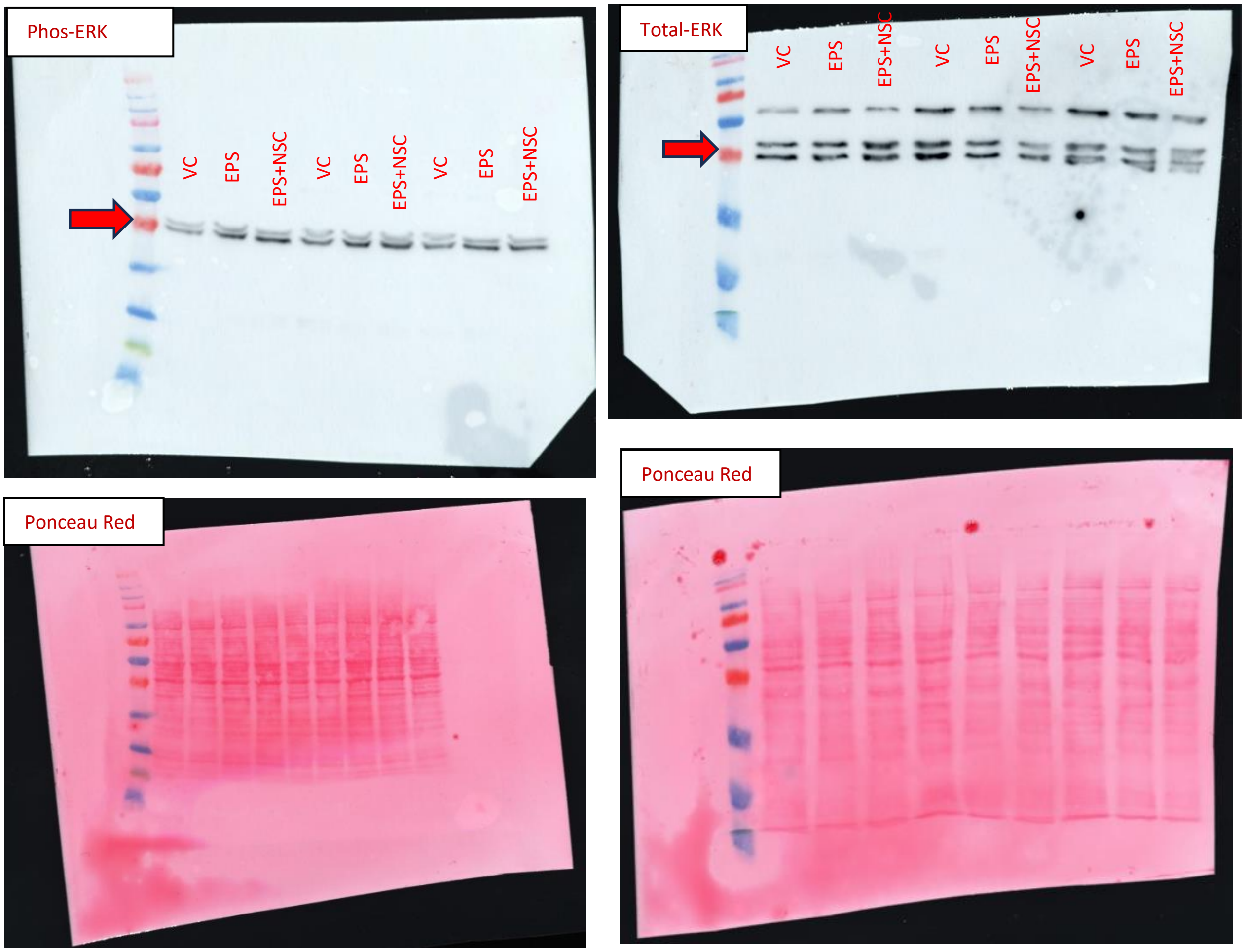

Supplemental Figure S1.C: Full images of phosphorylated ERK (left) and total ERK (Right) in the EPS condition, without (EPS) or with (EPS+NSC) NSC185058 treatment. Full Ponceau S stains are presented below each image.

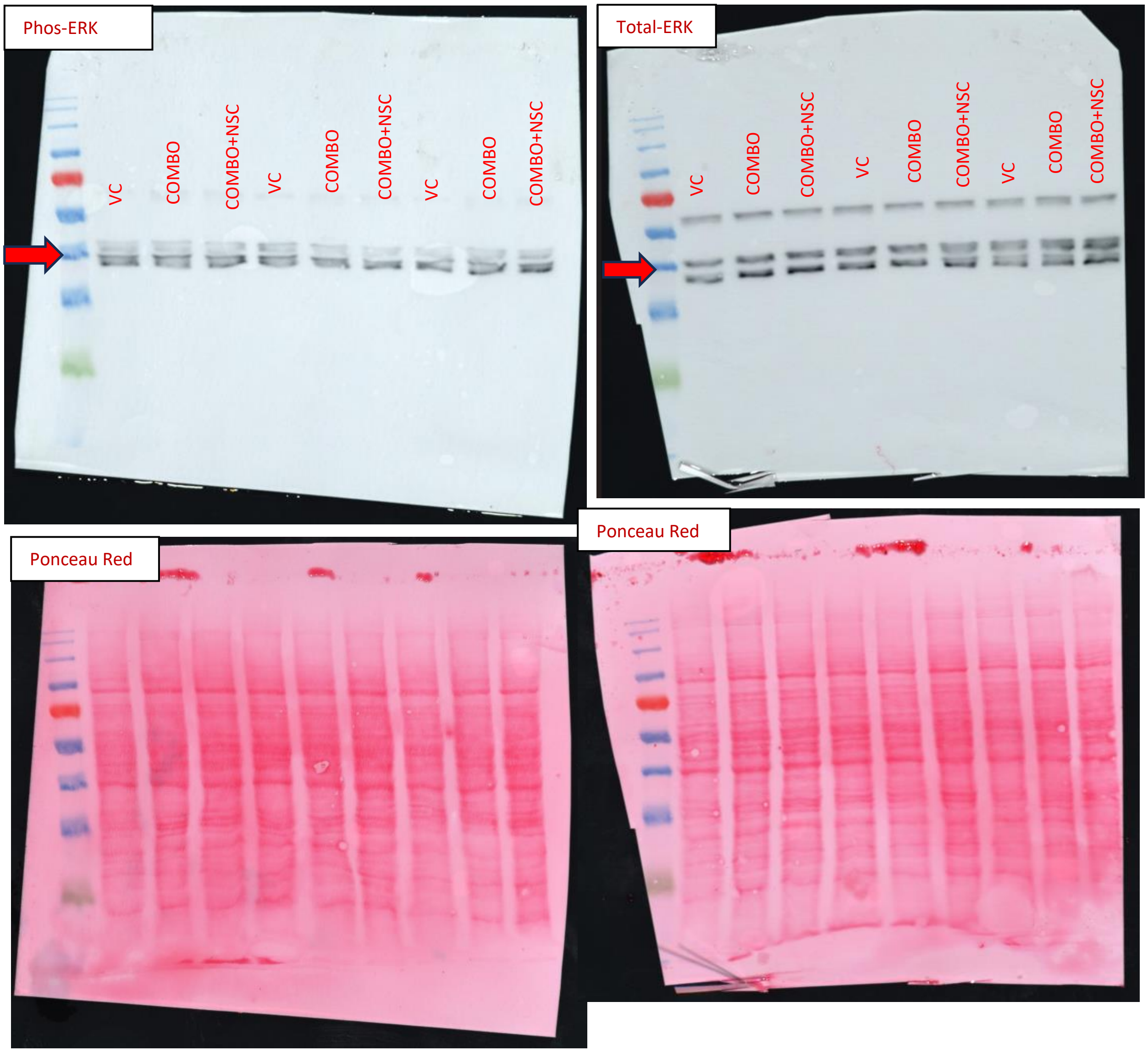

Supplemental Figure S1.D: Full images of phosphorylated ERK (left) and total ERK (Right) in the Combo condition, without (Combo) or with (Combo+NSC) NSC185058 treatment. Full Ponceau S stains are presented below each image.

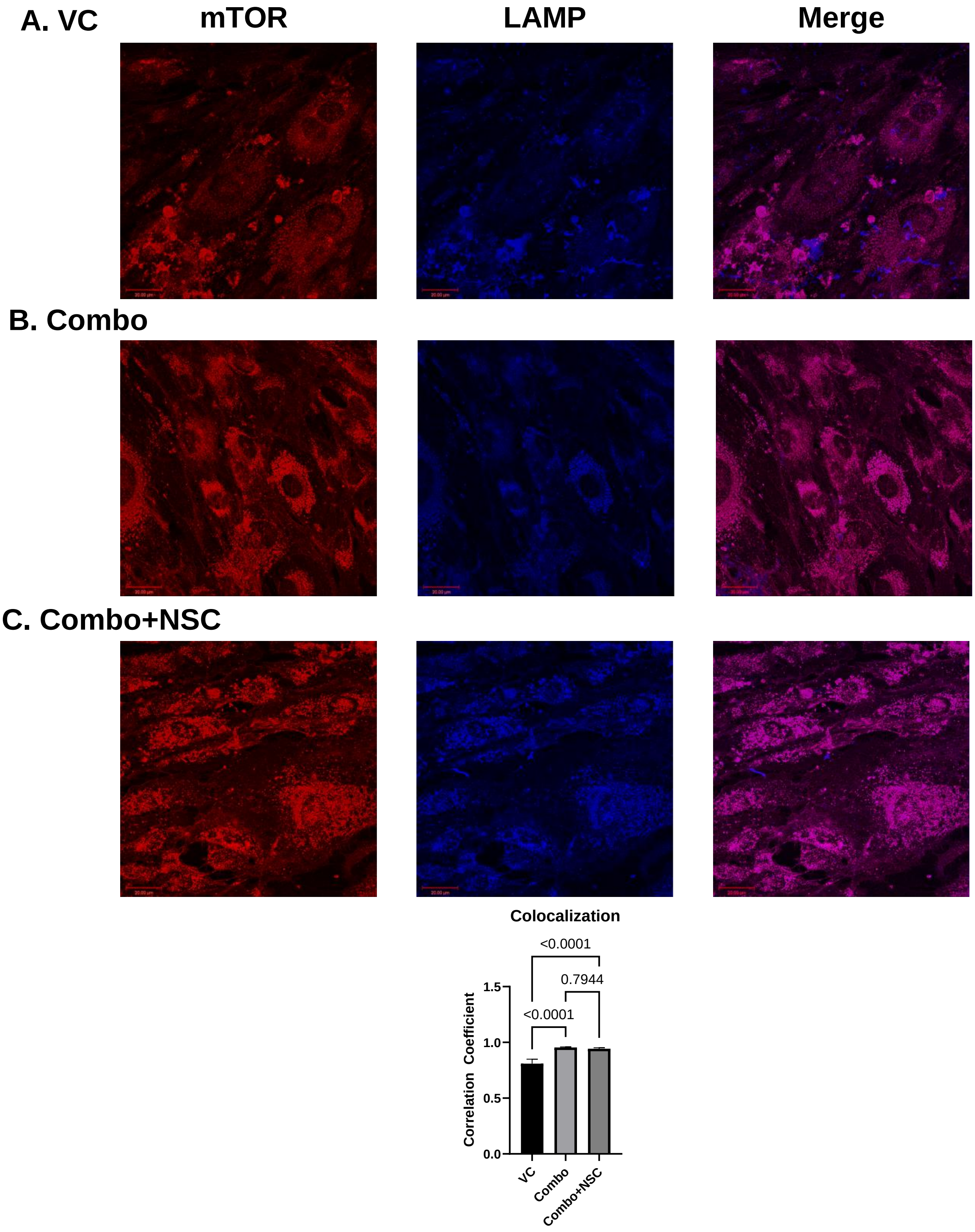
A. VC
mTOR
LAMP
Merge
B. Combo
C. Combo+NSC
Colocalization
<0.0001
0.7944
<0.0001
Correlation Coefficient
1.5
1.0
0.5
0.0
VC
Combo
Combo+NSC

Supplemental Figure S2: NSC185058 Does Not Affect mTOR Translocation to the Lysosome. Representative confocal microscope images showing that mTOR translocation to the lysosome in response to the Combo condition is unaffected by NSC185058 (NSC) versus vehicle control (VC) (n=4).

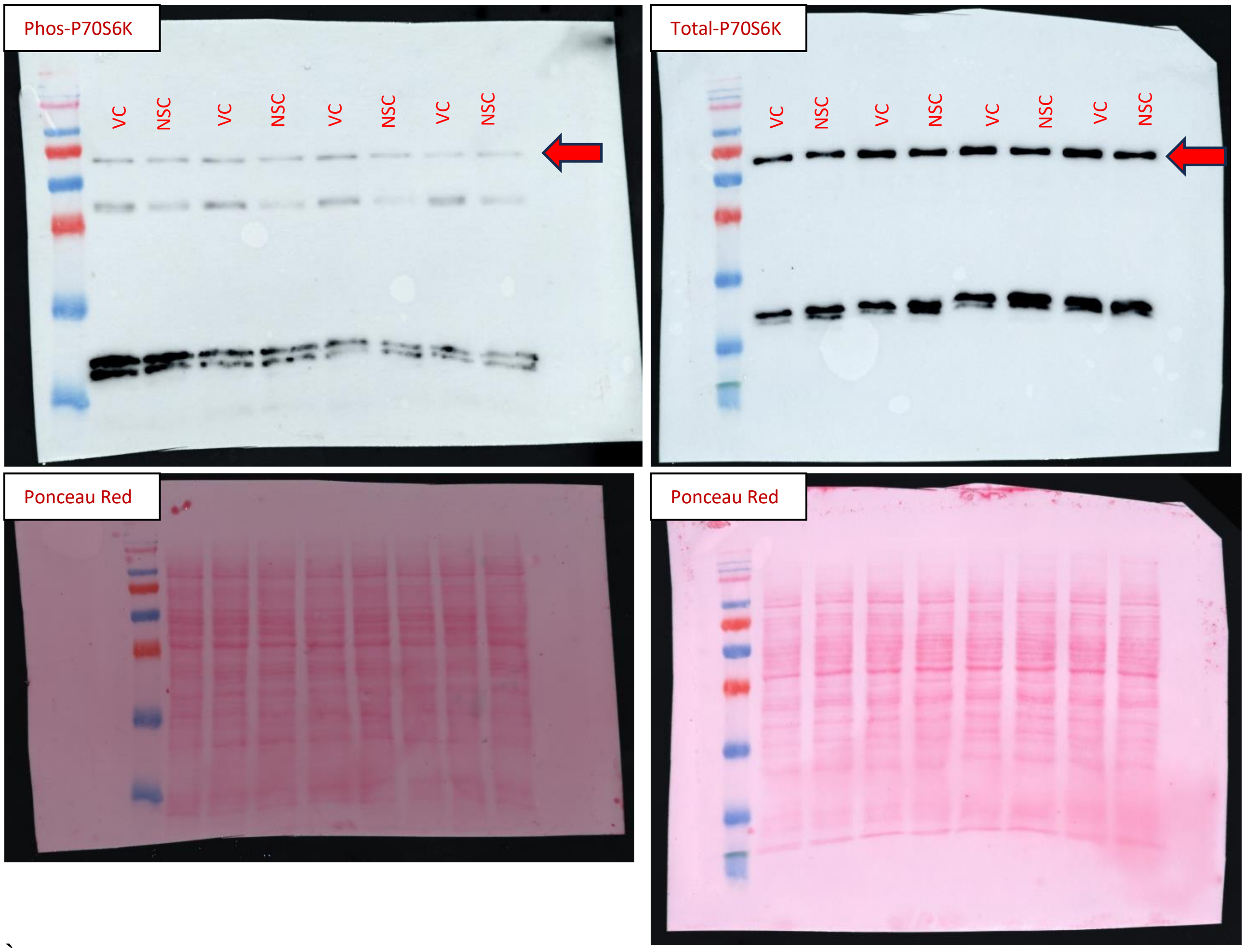

`

Supplemental Figure S3.A: Full images of phosphorylated P70S6K (left) and total P70S6K (Right) in the Baseline condition, without (VC) or with (NSC) NSC185058 treatment. Full Ponceau S stains are presented below each image.

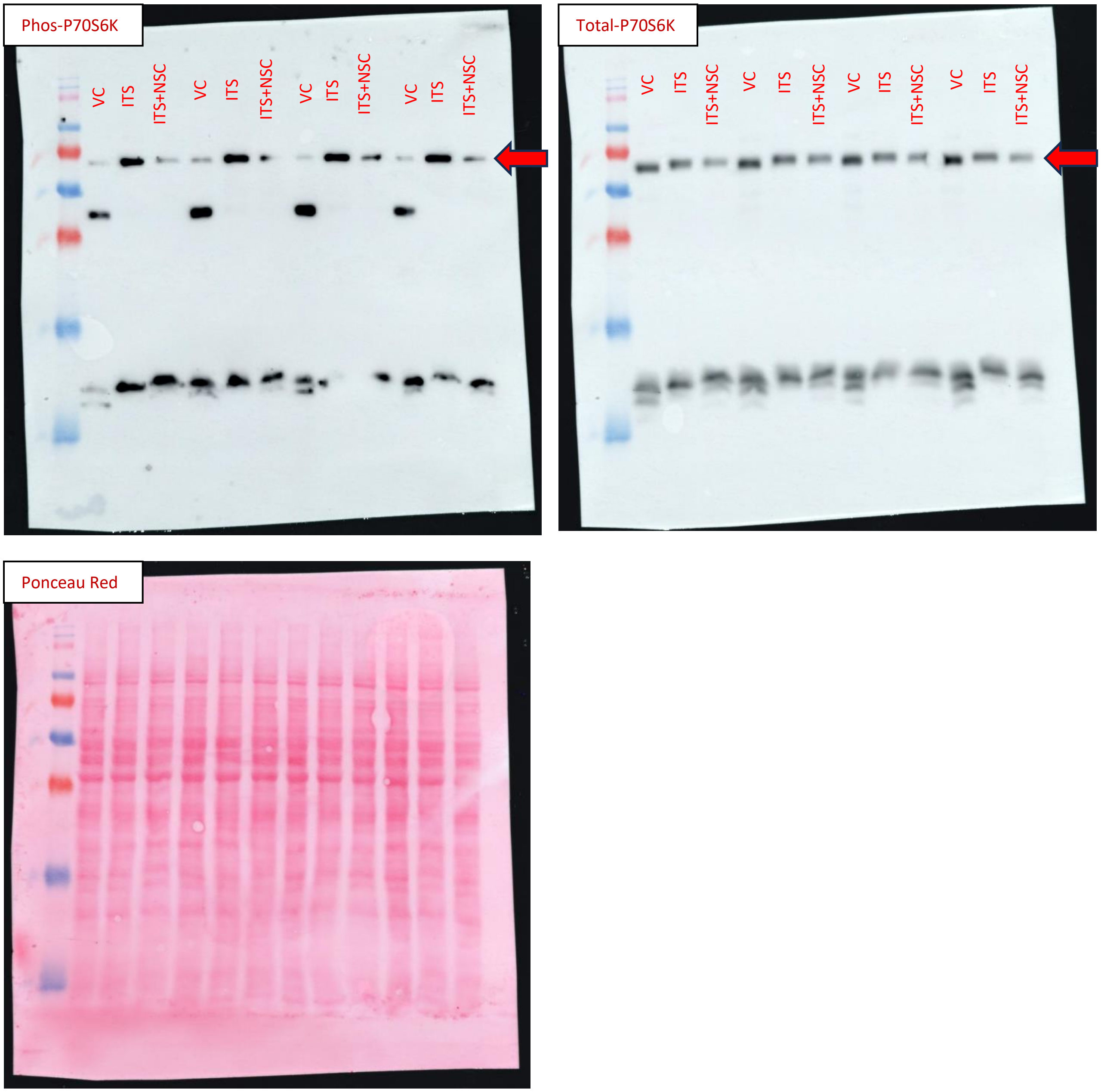

Supplemental Figure S3.B: Full images of phosphorylated P70S6K (left) and total P70S6K (Right) in the ITS condition, without (ITS) or with (ITS+NSC) NSC185058 treatment. In this case membrane was stripped and re-probed, so only one Ponceau S stain is presented.

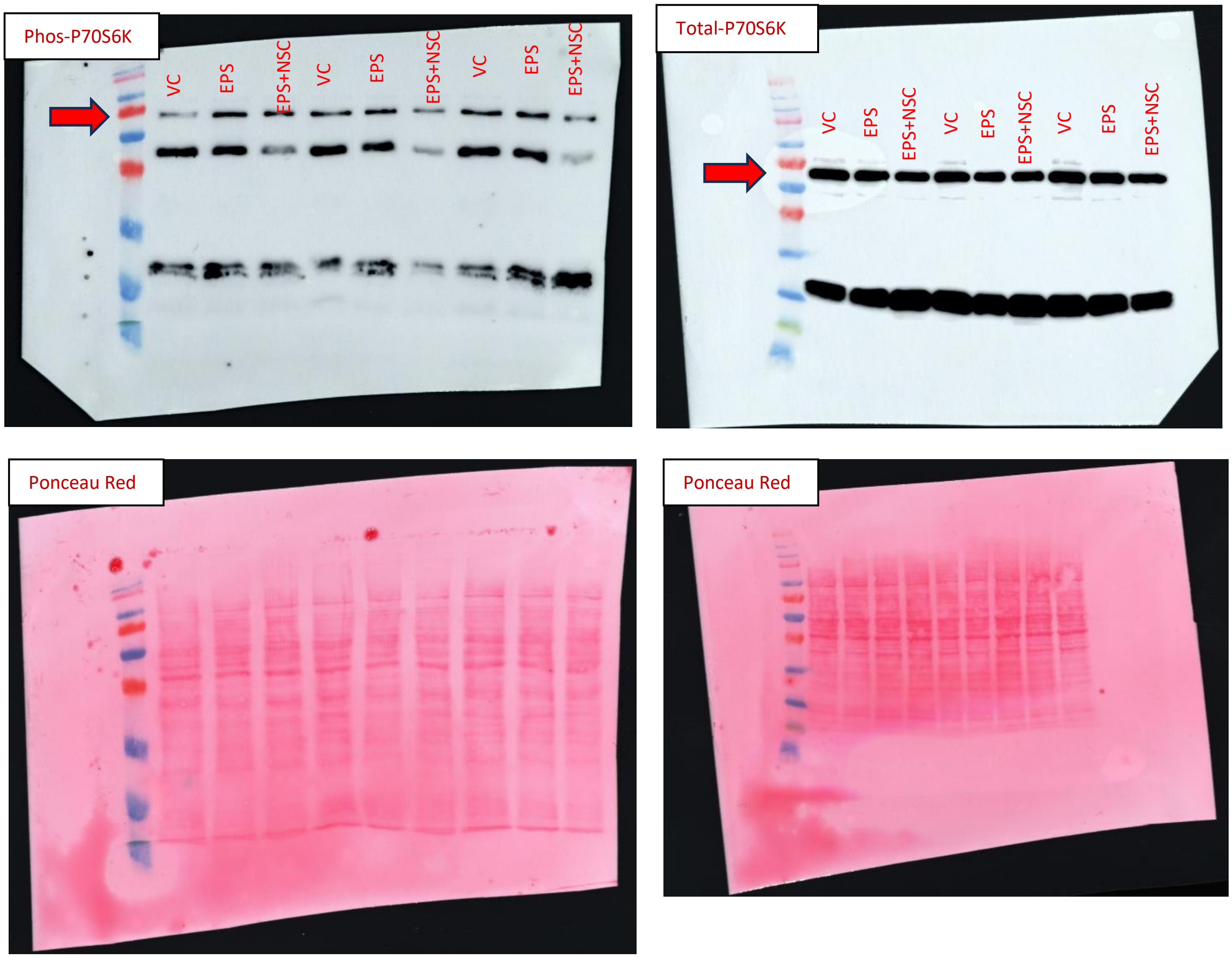

Supplemental Figure S3.C: Full images of phosphorylated P70S6K (left) and total P70S6K (Right) with EPS treatment. Full Ponceau S stains are presented below each image.

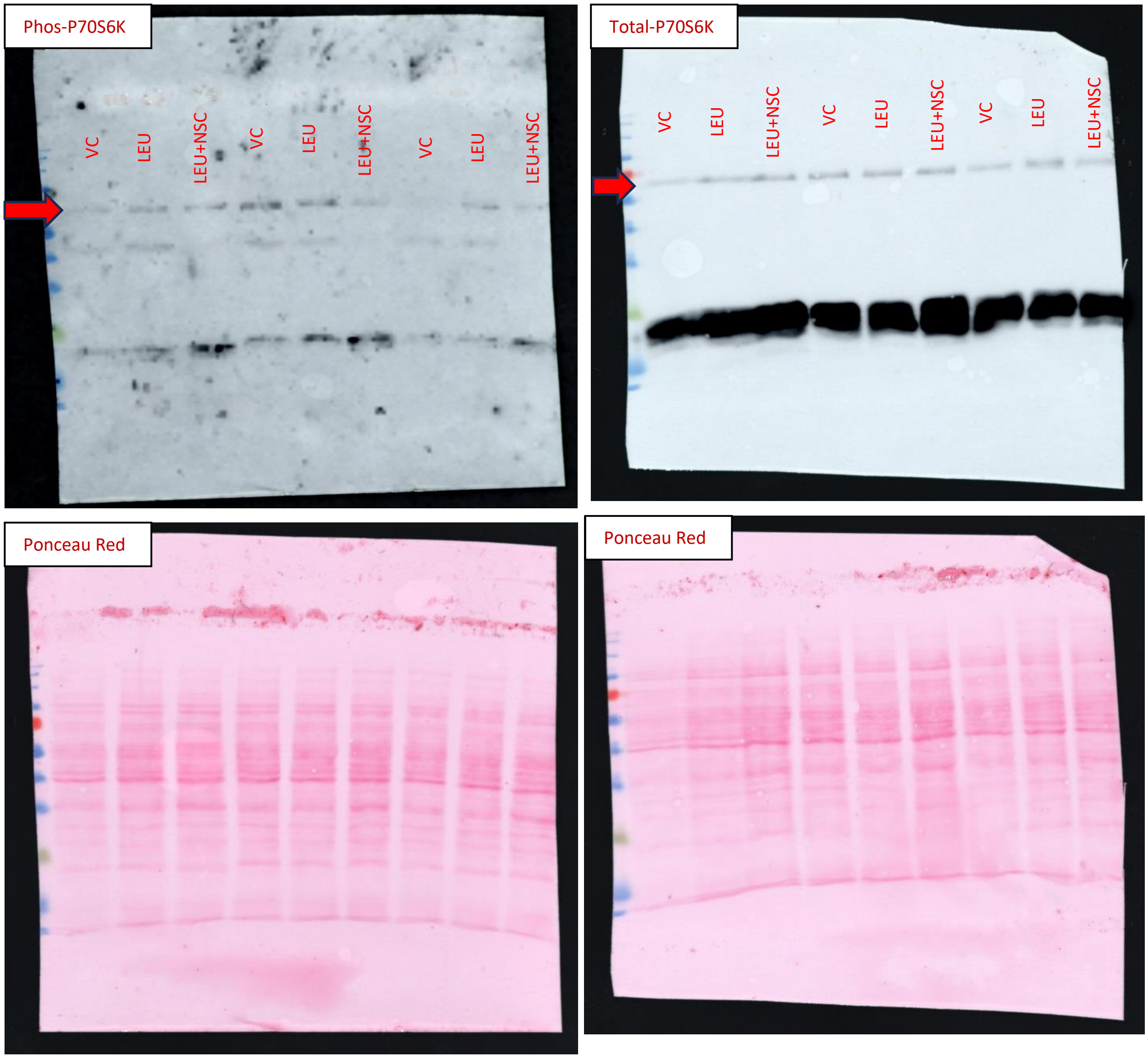

Supplemental Figure S3.D: Full images of phosphorylated P70S6K (left) and total P70S6K (Right) in the LEU condition, without (LEU) or with (LEU+NSC) NSC185058 treatment. Full Ponceau S stains are presented below each image.

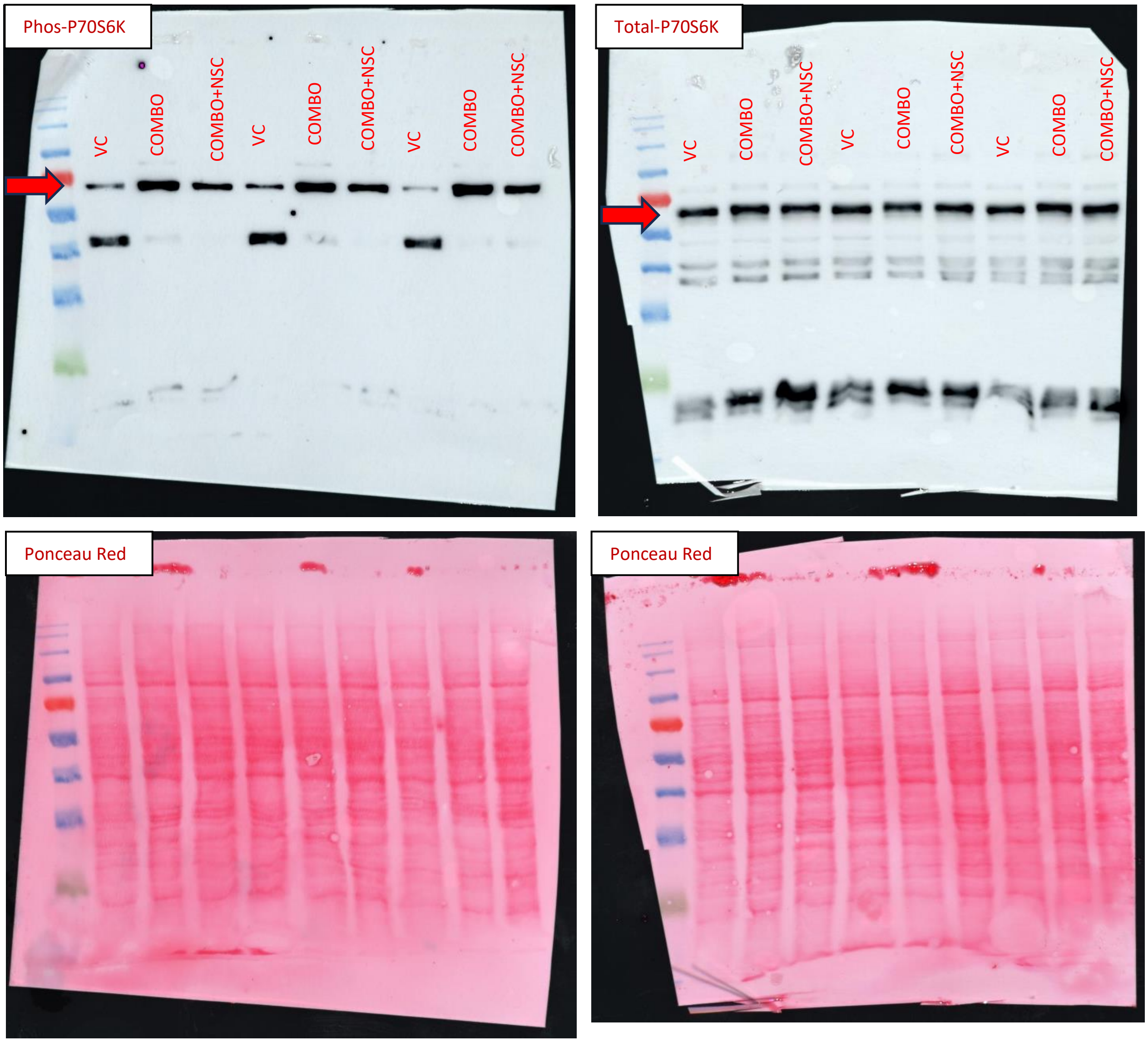

Supplemental Figure S3.E: Full images of phosphorylated P70S6K (left) and total P70S6K (Right) in the Combo condition, without (Combo) or with (Combo+NSC) NSC185058 treatment. Full Ponceau S stains are presented below each image.

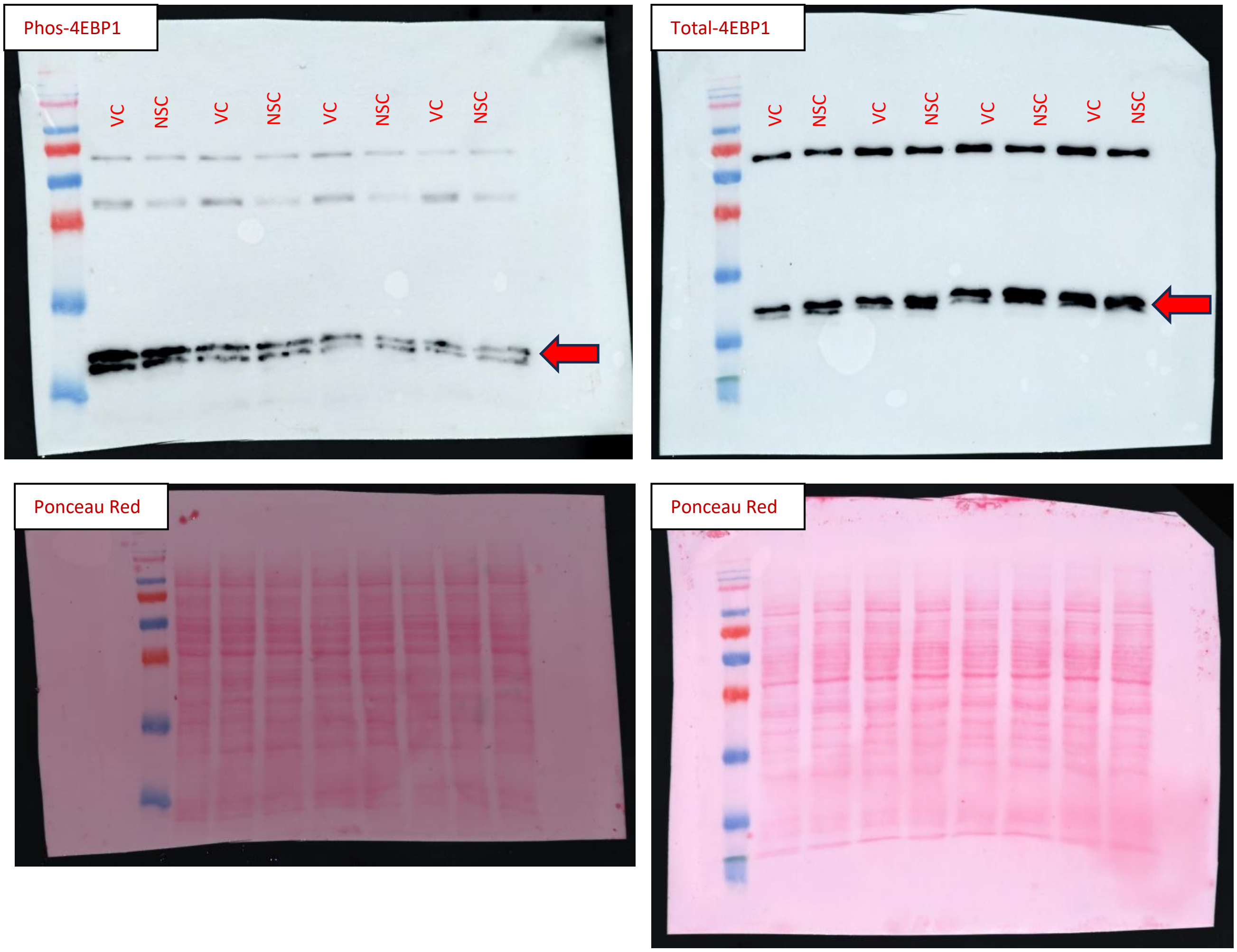

Supplemental Figure S3.F: Full images of phosphorylated 4EBP1 (left) and total 4EBP1 (Right) in the Baseline condition, without (VC) or with (NSC) NSC185058 treatment. Full Ponceau S stains are presented below each image.

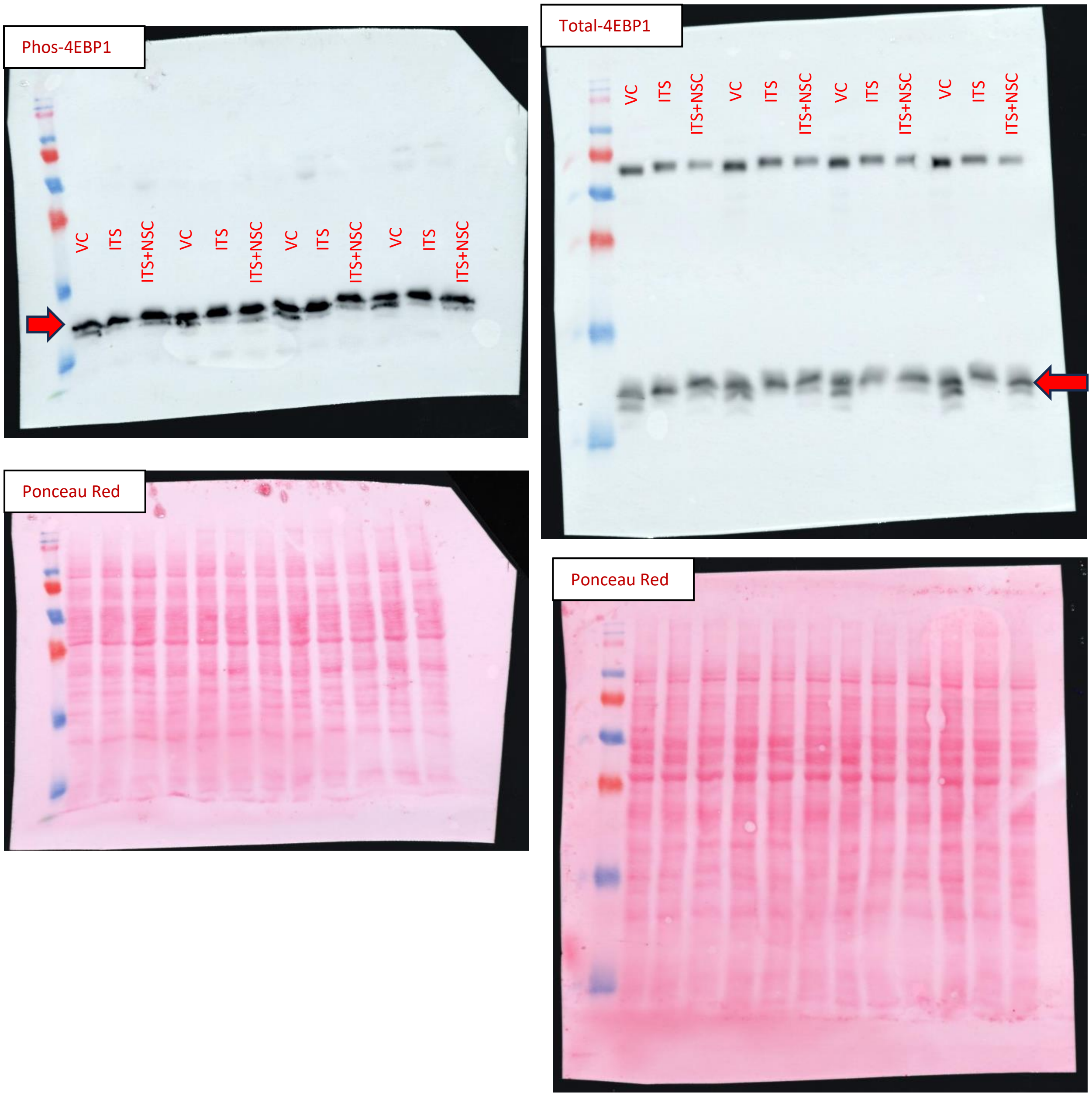

Supplemental Figure S3.G: Full images of phosphorylated 4EBP1 (left) and total 4EBP1 (Right) in the ITS condition, without (ITS) or with (ITS+NSC) NSC185058 treatment. Full Ponceau S stains are presented below each image.

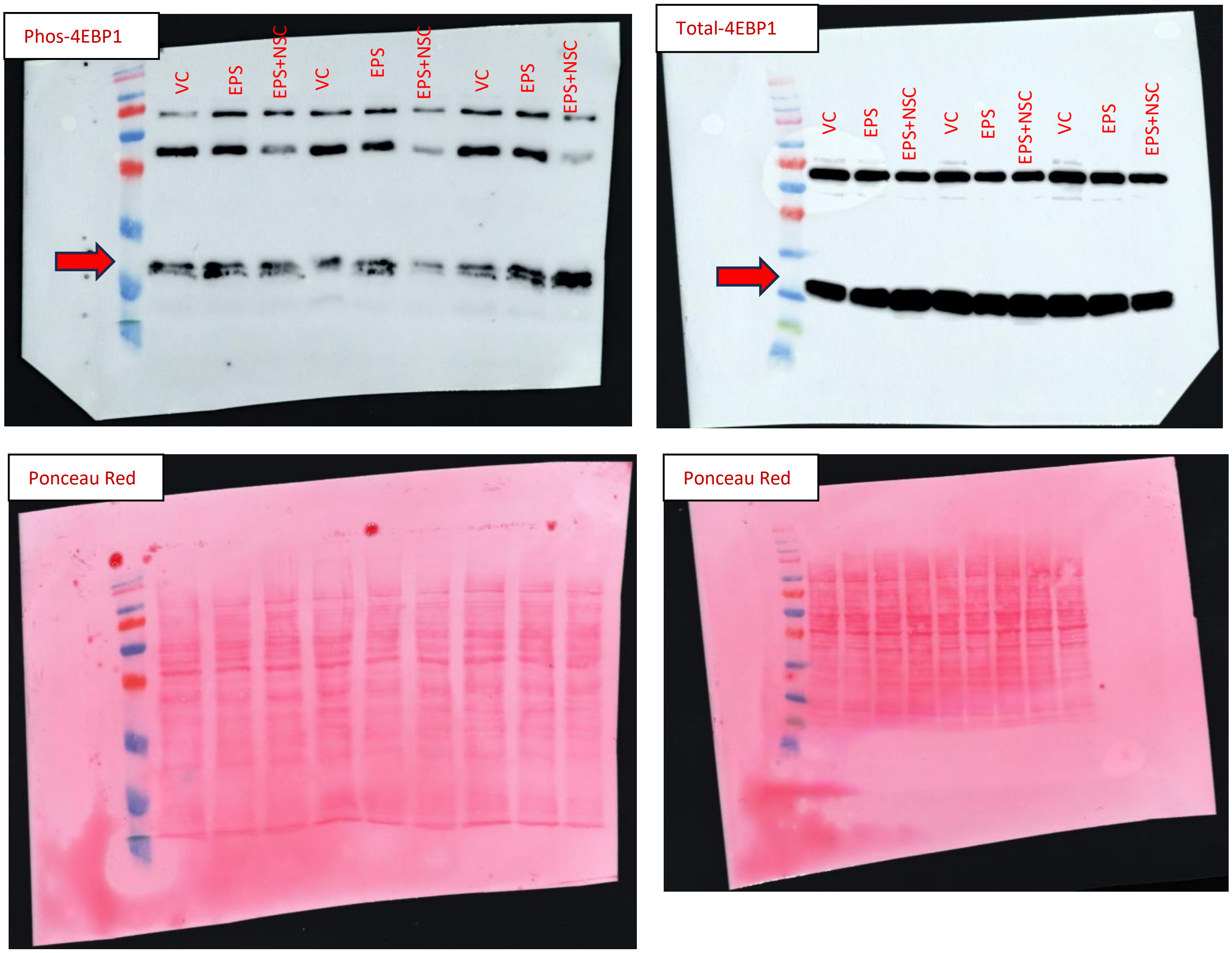

Supplemental Figure S3.H: Full images of phosphorylated 4EBP1 (left) and total 4EBP1 (Right) in the EPS condition, without (EPS) or with (EPS+NSC) NSC185058 treatment. Full Ponceau S stains are presented below each image.

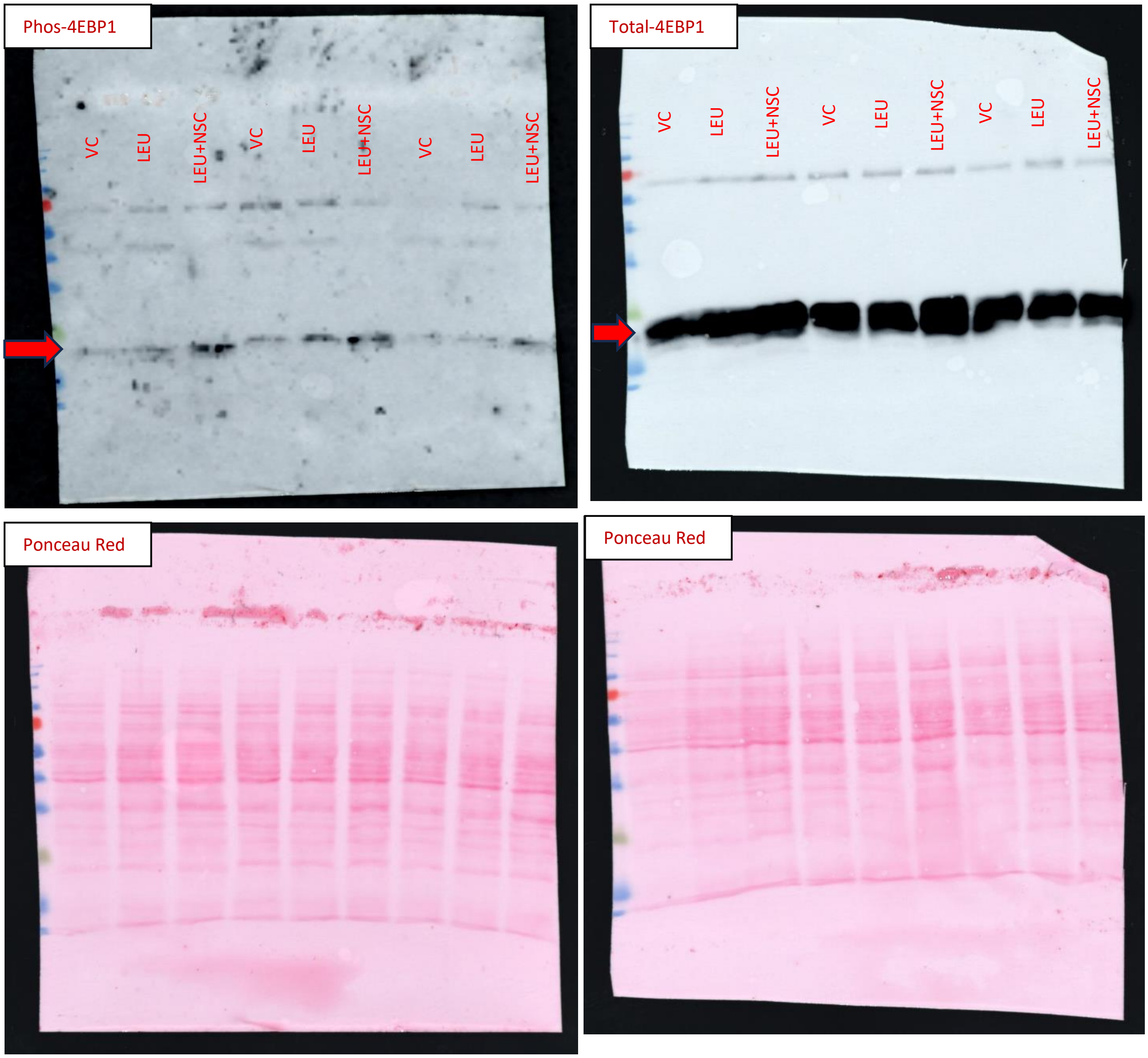

Supplemental Figure S3.I: Full images of phosphorylated 4EBP1 (left) and total 4EBP1 (Right) in the LEU condition, without (LEU) or with (LEU+NSC) NSC185058 treatment. Full Ponceau S stains are presented below each image.

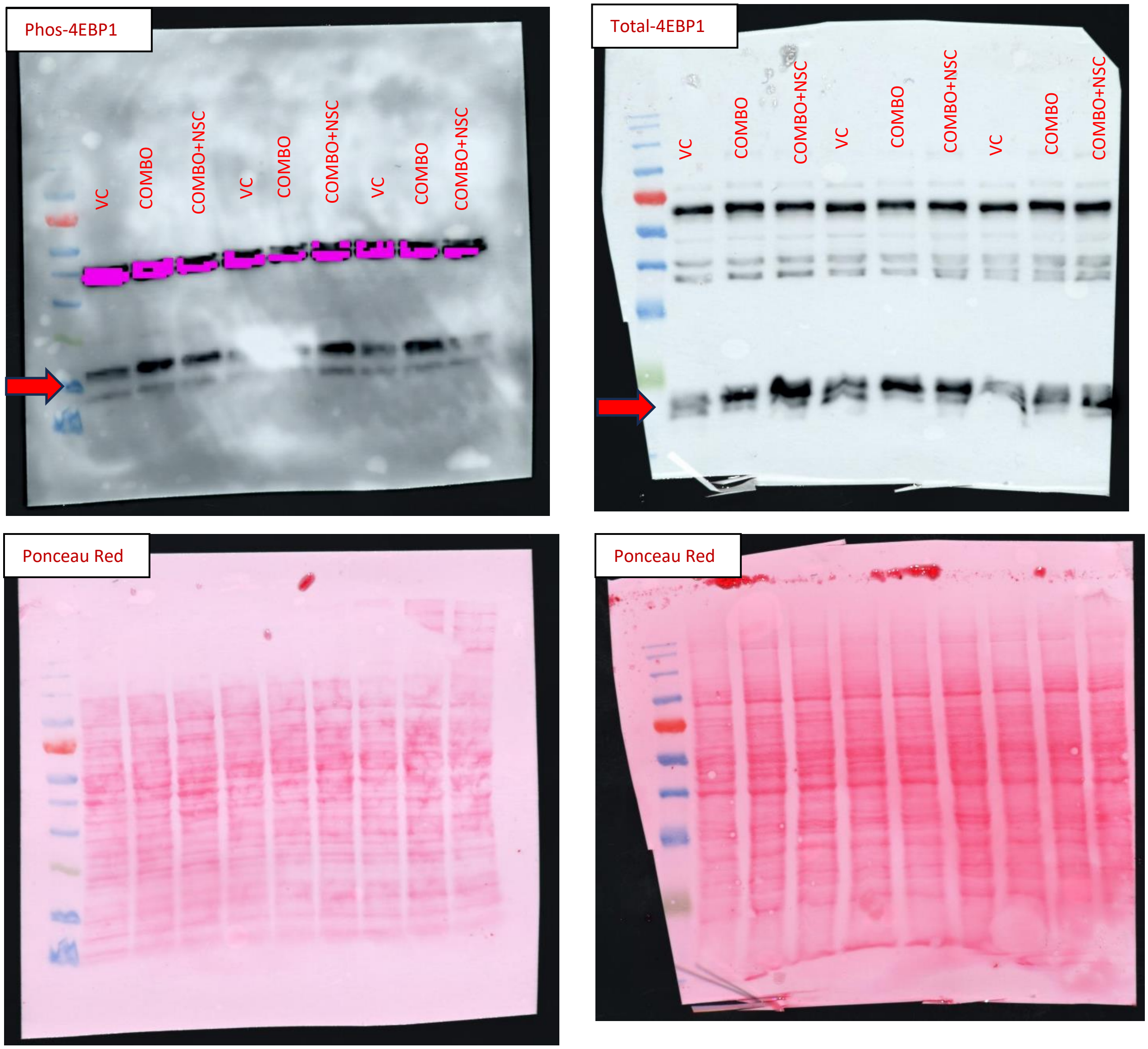

Supplemental Figure S3.J: Full images of phosphorylated 4EBP1 (left) and total 4EBP1 (Right) in the Combo condition, without (COMBO) or with (COMBO+NSC) NSC185058 treatment. Full Ponceau S stains are presented below each image.

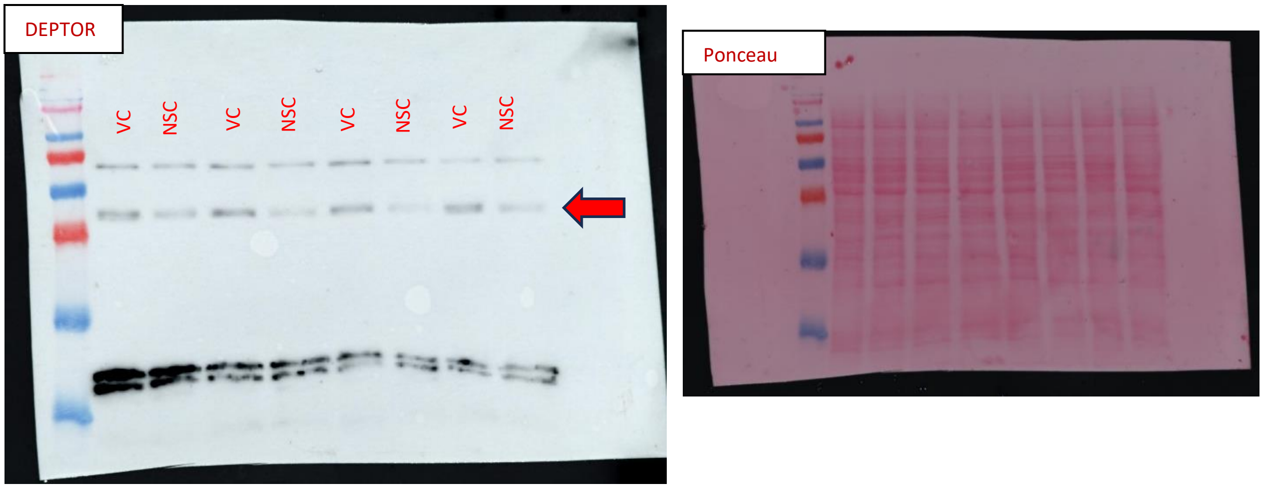

Supplemental Figure S4.A: Full image of DEPTOR in the Baseline condition, without (VC) or with (NSC) NSC185058 treatment. Full Ponceau S stain is presented to the right.

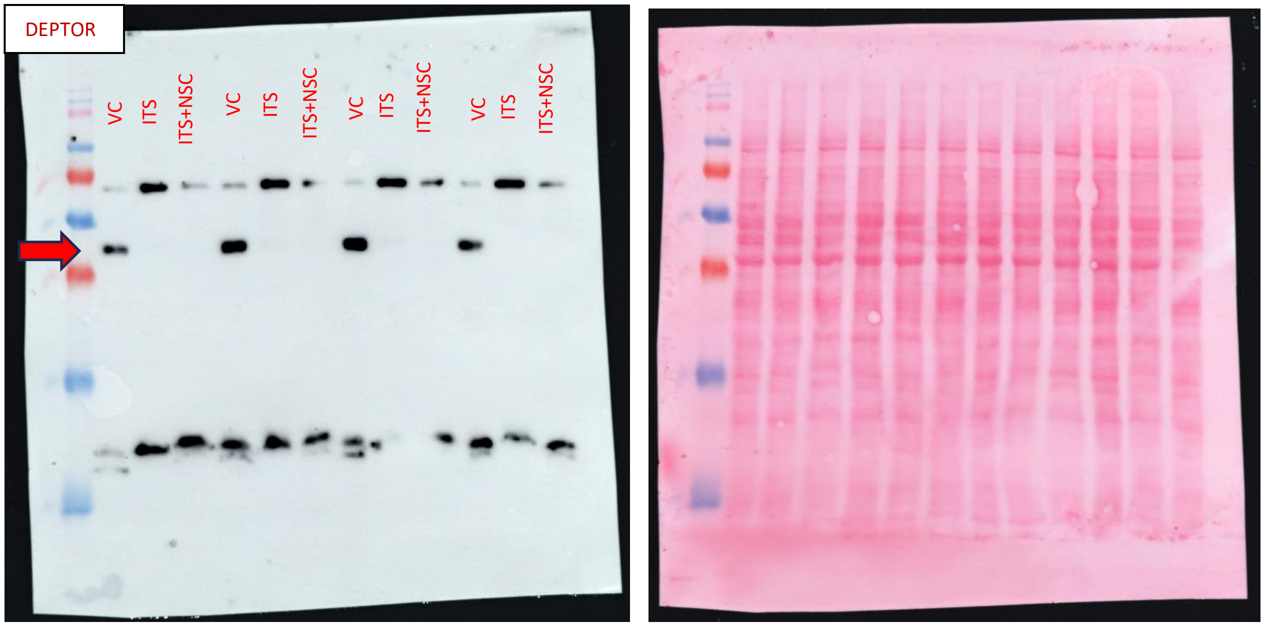

Supplemental Figure S4.B: Full image of DEPTOR in the ITS condition, without (ITS) or with (ITS+NSC) NSC185058 treatment. Full Ponceau S stain is presented to the right.

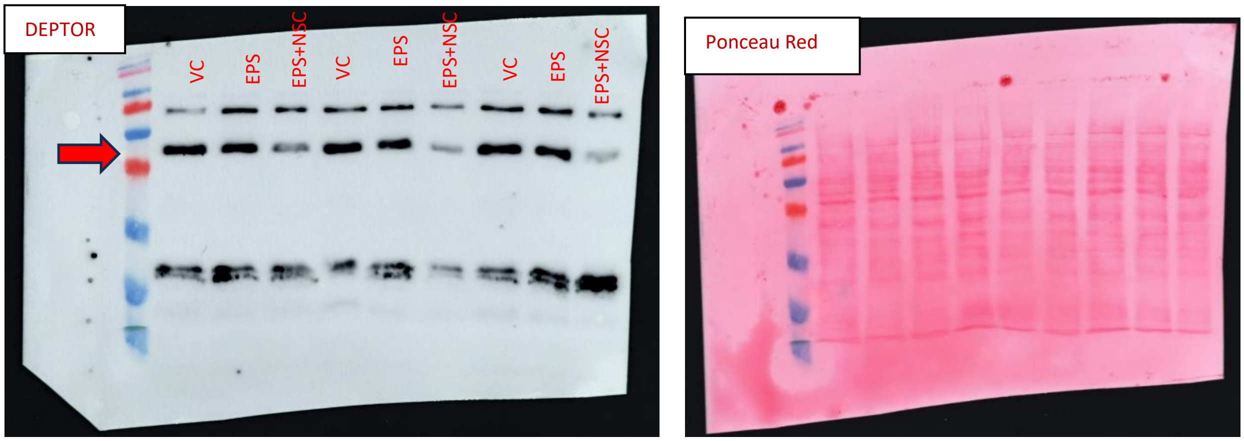

Supplemental Figure S4.C: Full image of DEPTOR in the EPS condition, without (EPS) or with (EPS+NSC) NSC185058 treatment. Full Ponceau S stain is presented to the right.

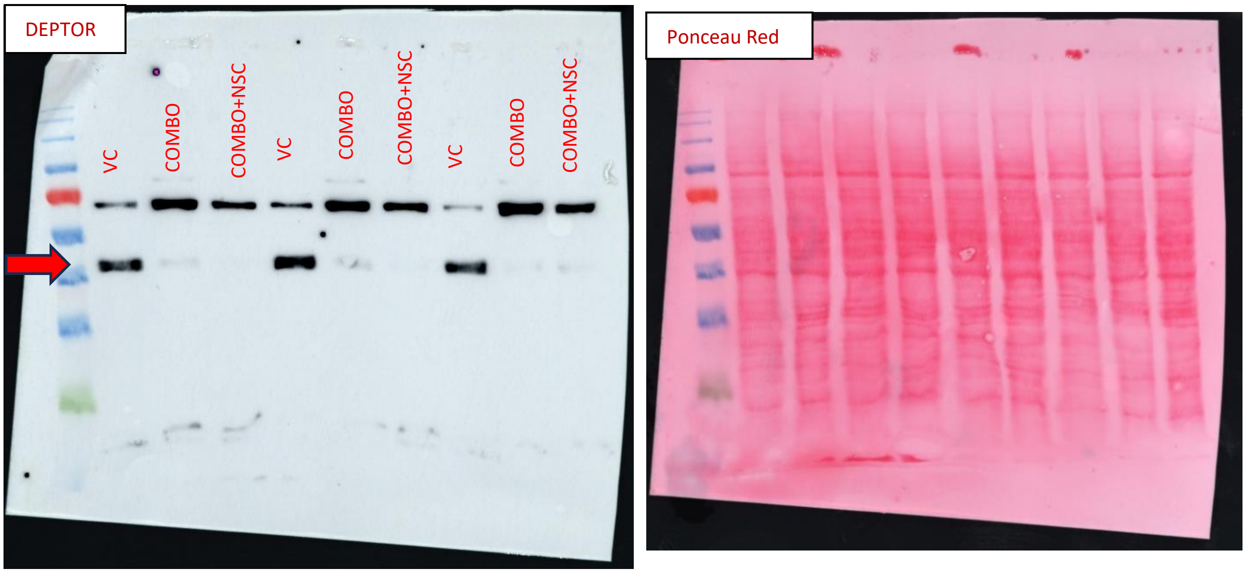

Supplemental Figure S4.D: Full image of DEPTOR in the Combo condition, without (COMBO) or with (COMBO+NSC) NSC185058 treatment. Full Ponceau S stain is presented to the right.

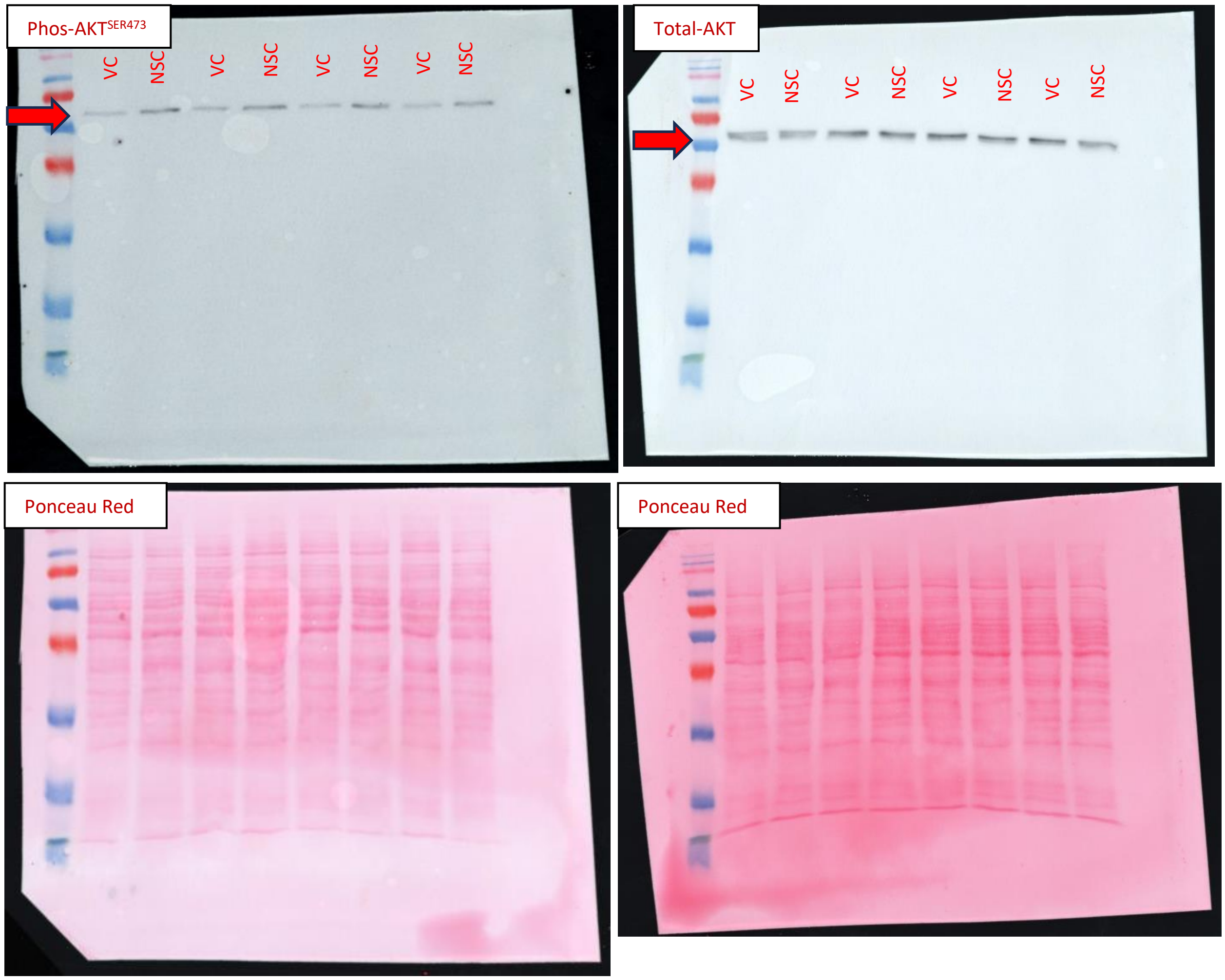

Supplemental Figure S4.E: Full images of phosphorylated AKT^{SER473} (left) and total AKT (right) in the Baseline condition, without (VC) or with (NSC) NSC185058 treatment. Full Ponceau S stains are presented below each image.

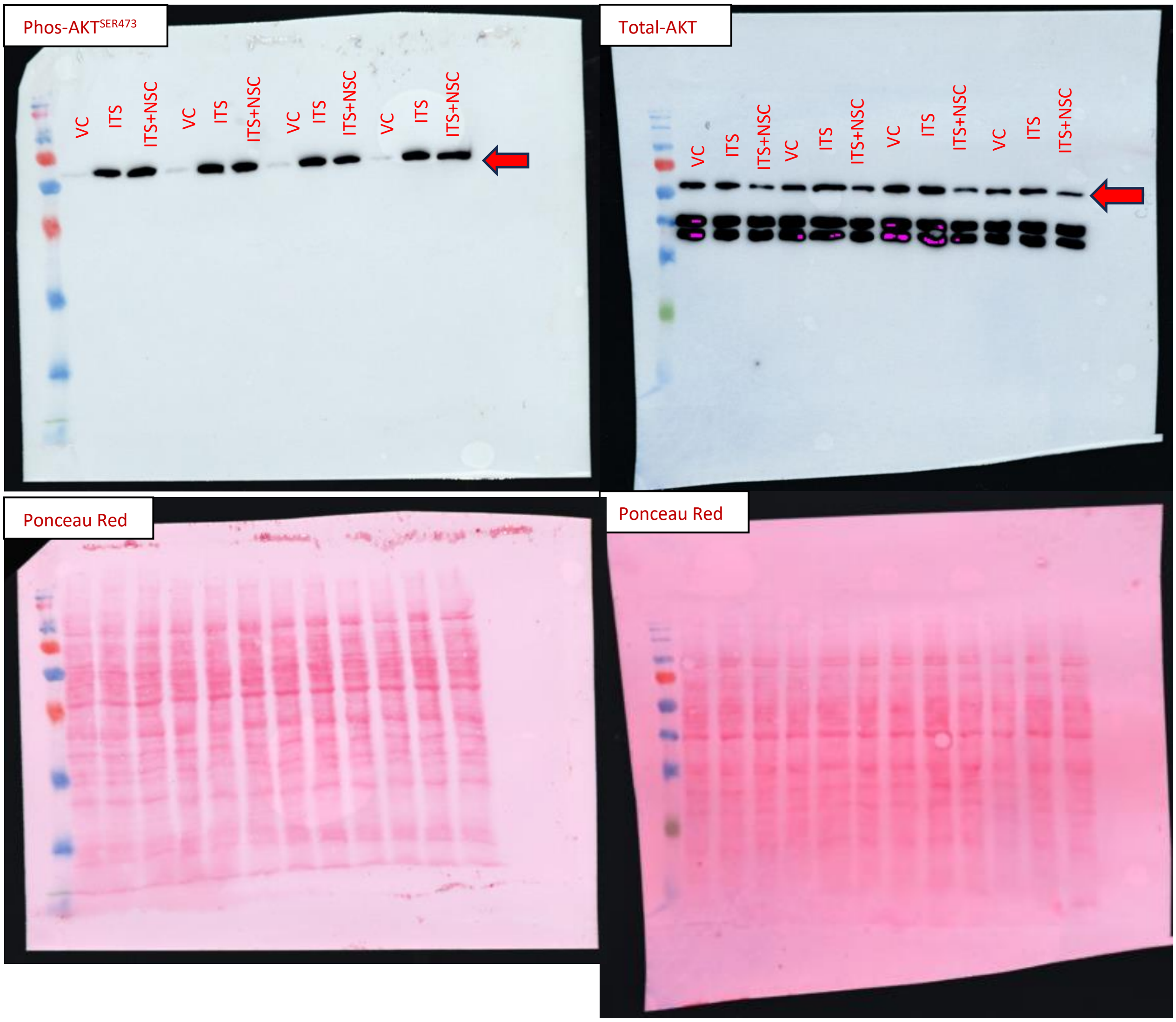

Supplemental Figure S4.F: Full images of phosphorylated AKT^{SER473} (left) and total AKT (right) in the ITS condition, without (ITS) or with (ITS+NSC) NSC185058 treatment. Full Ponceau S stains are presented below each image.

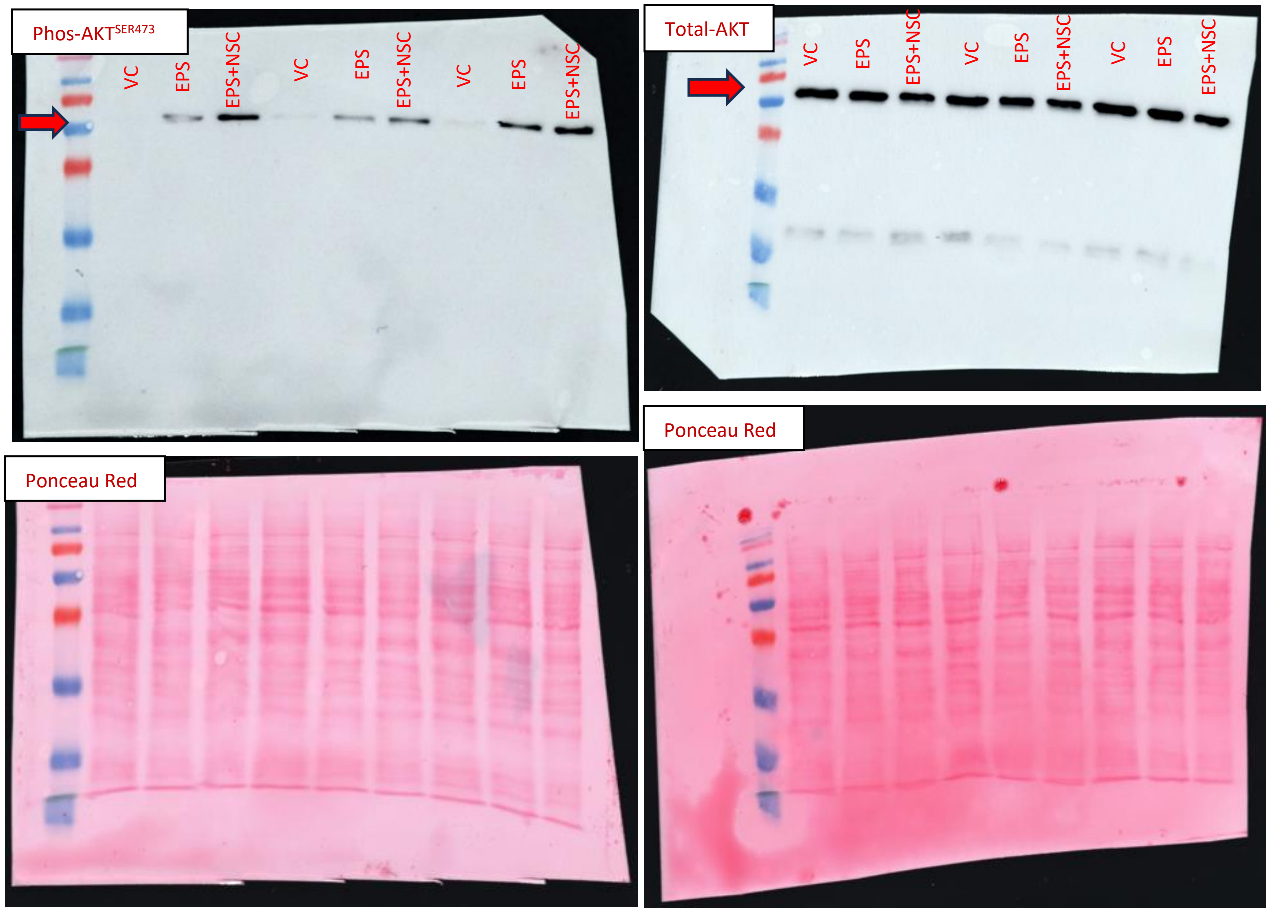

Supplemental Figure S4.G: Full images of phosphorylated AKT^{SER473} (left) and total AKT (right) in the EPS condition, without (EPS) or with (EPS+NSC) NSC185058 treatment. Full Ponceau S stains are presented below each image.

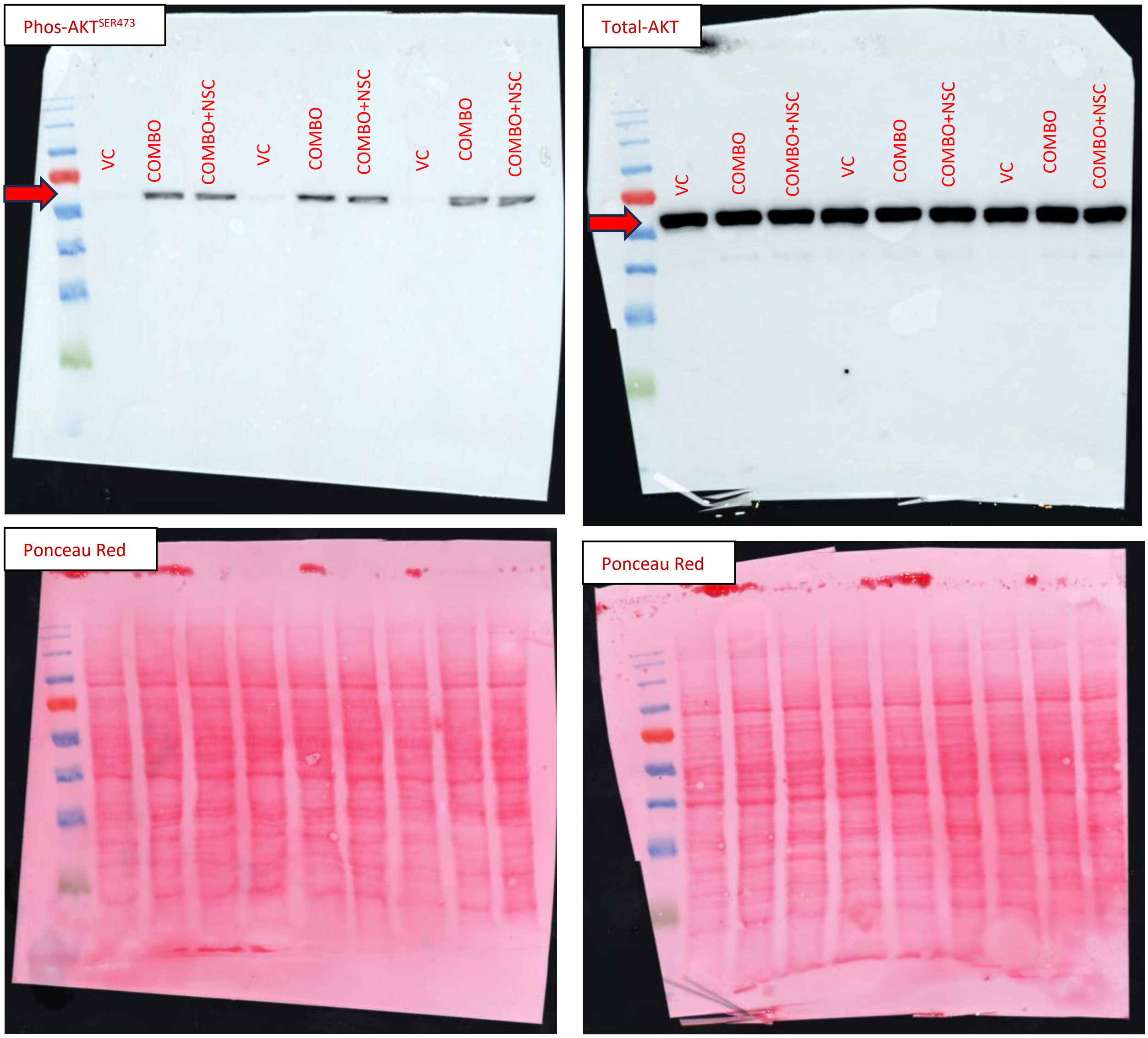

Supplemental Figure S4.H: Full images of phosphorylated AKT^{SER473} (left) and total AKT (right) in the Combo condition, without (COMBO) or with (COMBO+NSC) NSC185058 treatment. Full Ponceau S stains are presented below each image.

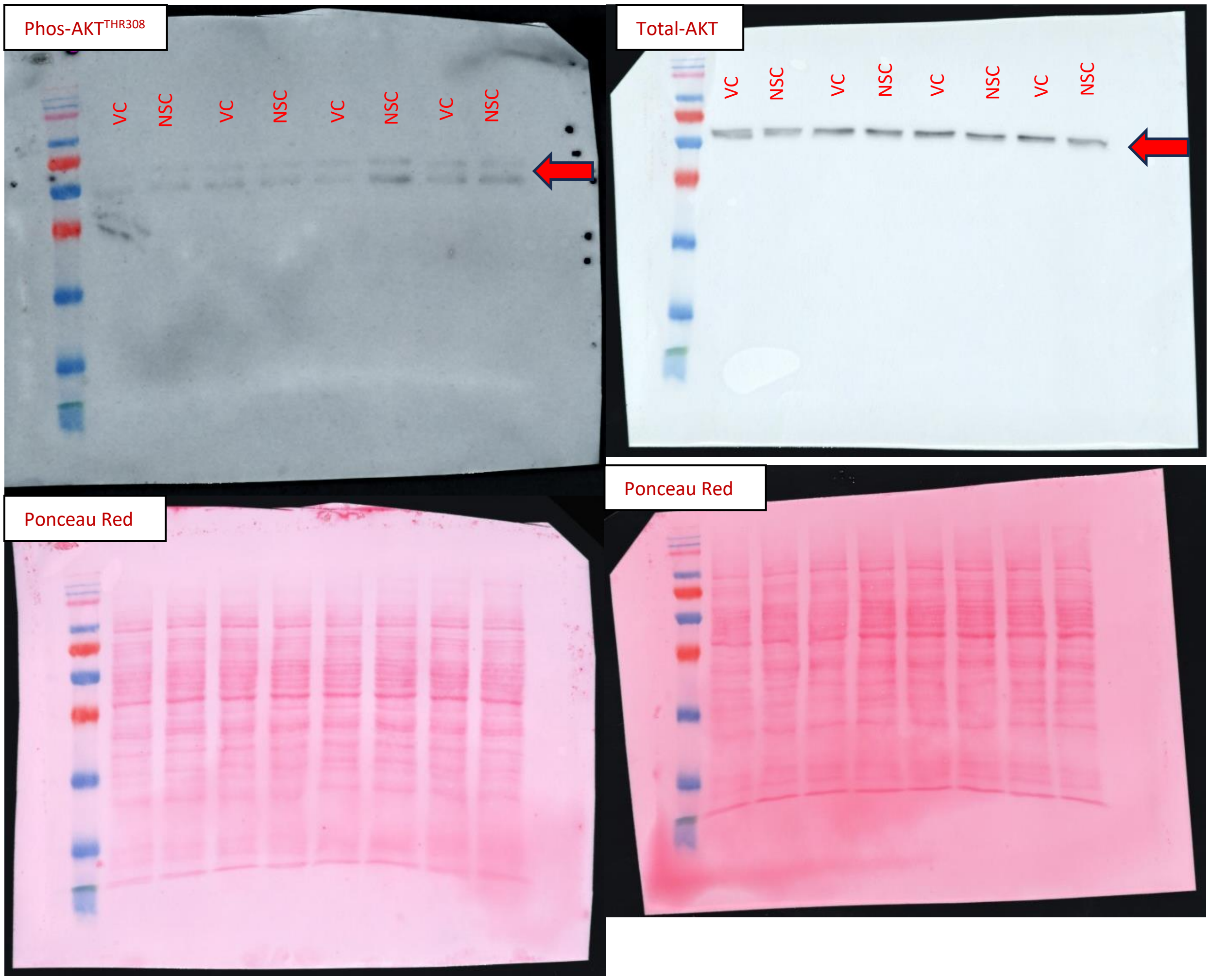

Supplemental Figure S4.I: Full images of phosphorylated AKT^{THR308} (left) and total AKT (right) in the Baseline condition, without (VC) or with (NSC) NSC185058 treatment. Full Ponceau S stains are presented below each image.

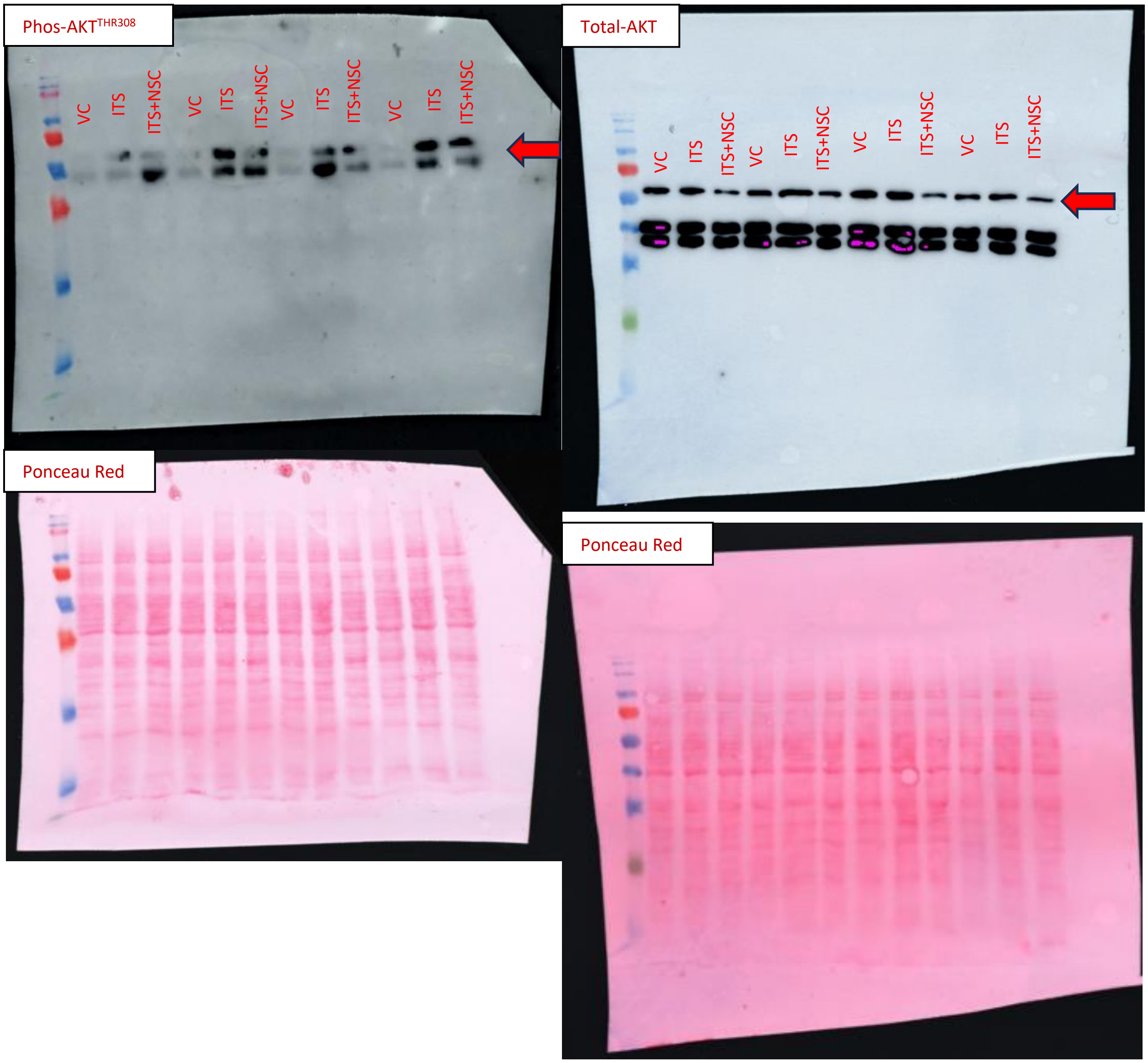

Supplemental Figure S4.J: Full images of phosphorylated AKT^{THR308} (left) and total AKT (right) in the ITS condition, without (ITS) or with (ITS+NSC) NSC185058 treatment. Full Ponceau S stains are presented below each image.

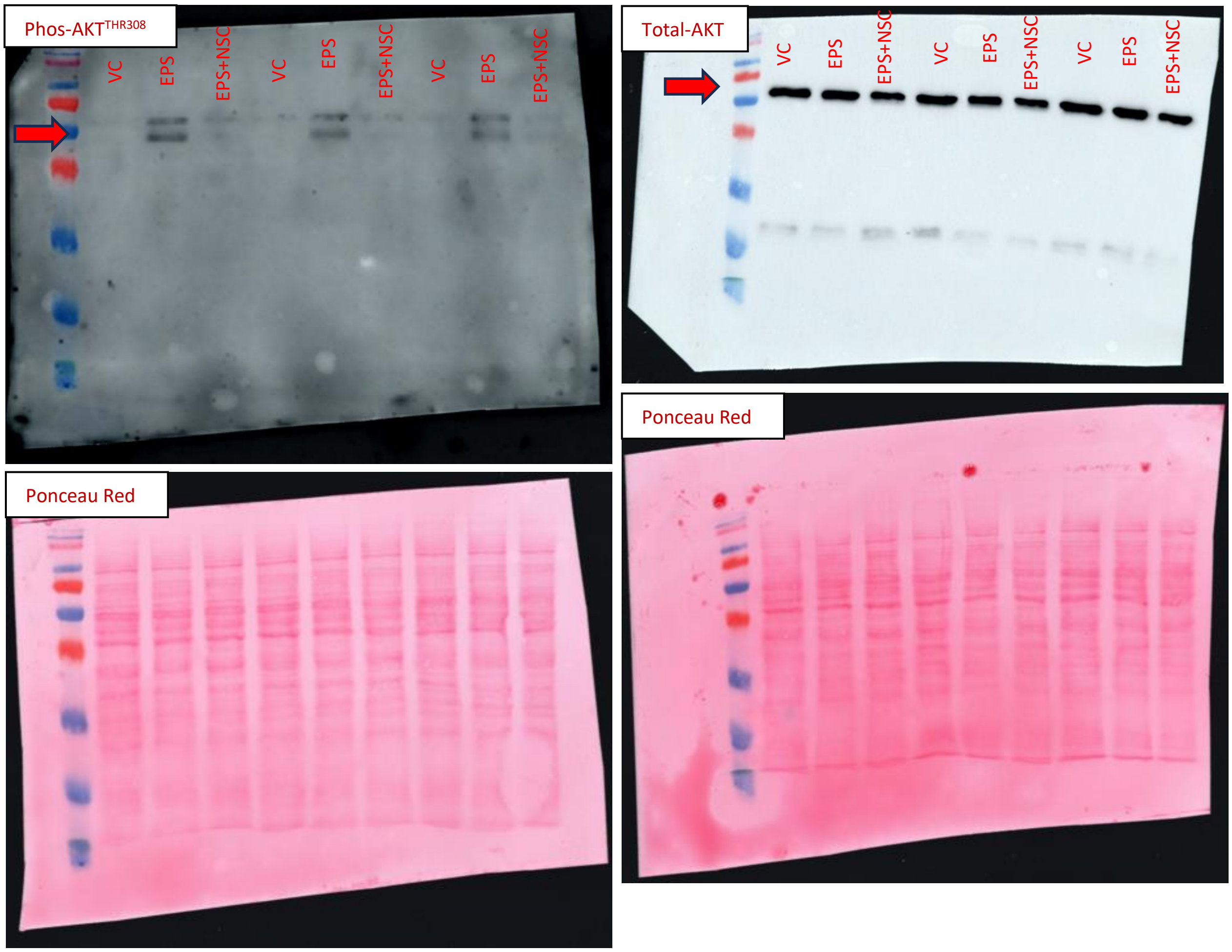

Supplemental Figure S4.K: Full images of phosphorylated AKT^{THR308} (left) and total AKT (right) in the EPS condition, without (EPS) or with (EPS+NSC) NSC185058 treatment. Full Ponceau S stains are presented below each image.

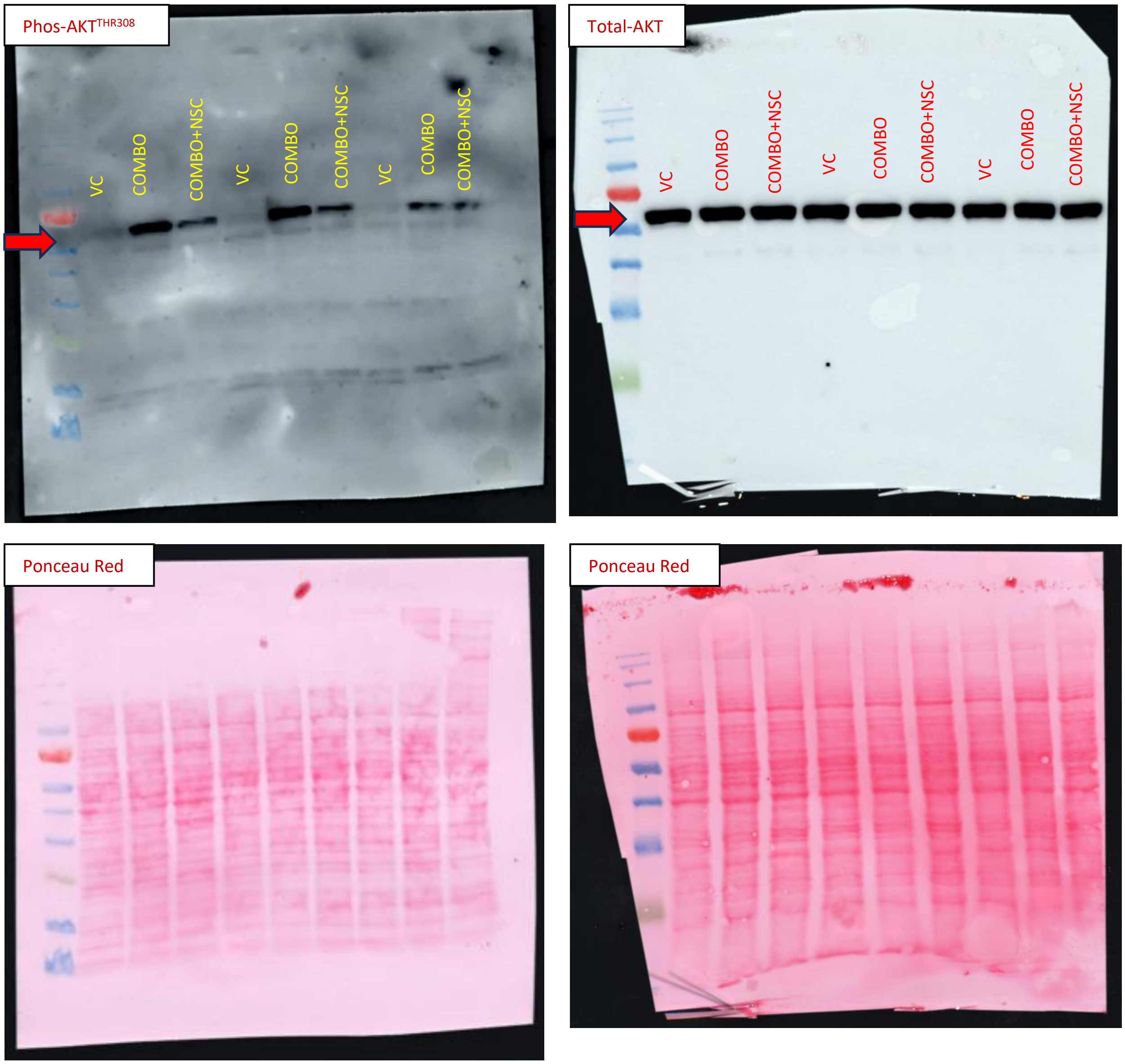

Supplemental Figure S4.L: Full images of phosphorylated AKT^{THR308} (left) and total AKT (right) in the Combo condition, without (Combo) or with (Combo+NSC) NSC185058 treatment. Full Ponceau S stains are presented below each image.

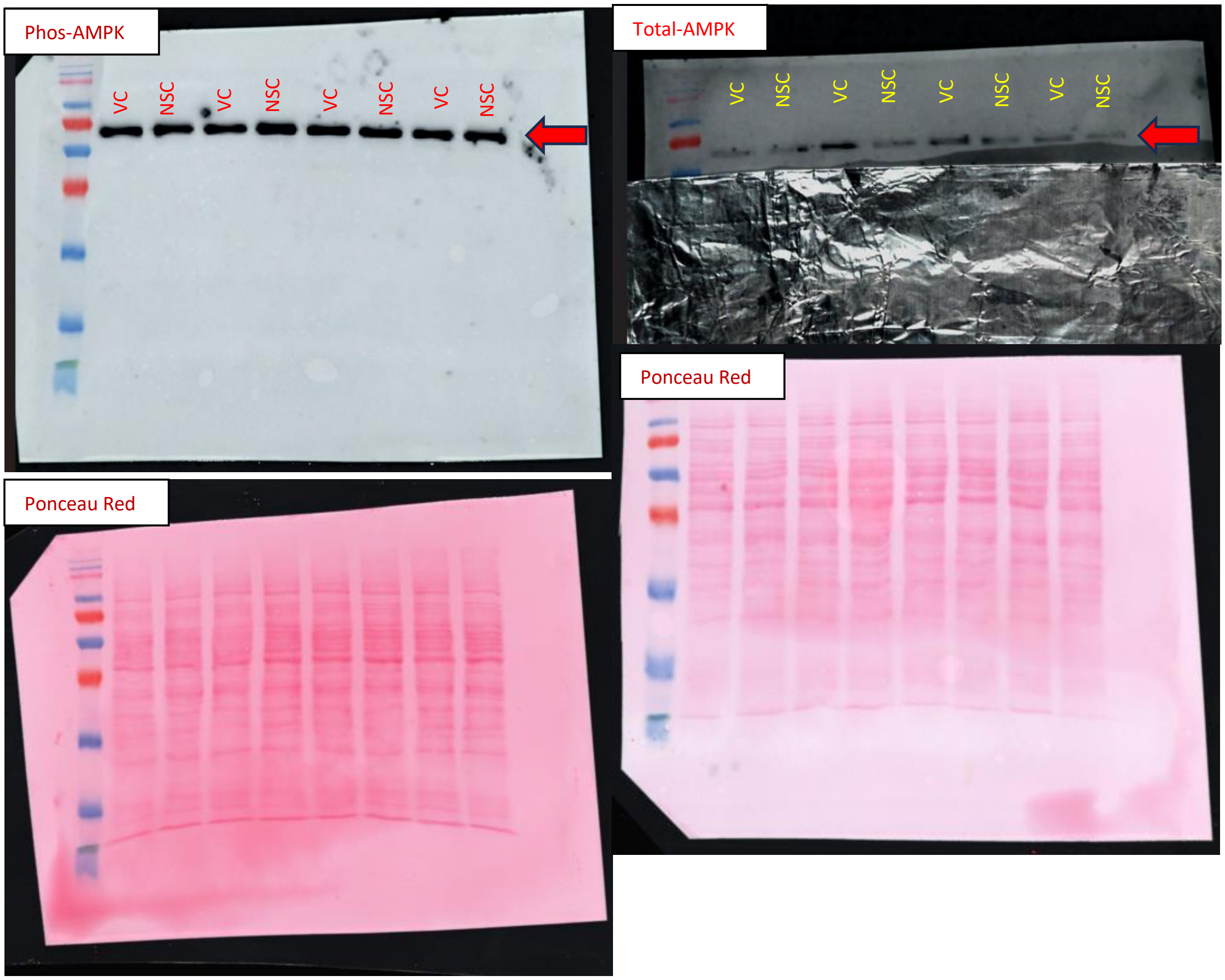

Supplemental Figure S5.E: Full images of phosphorylated AMPK (left) and total AMPK (right) in the Baseline condition, without (VC) or with (NSC) NSC185058 treatment. Full Ponceau S stains are presented below each image.

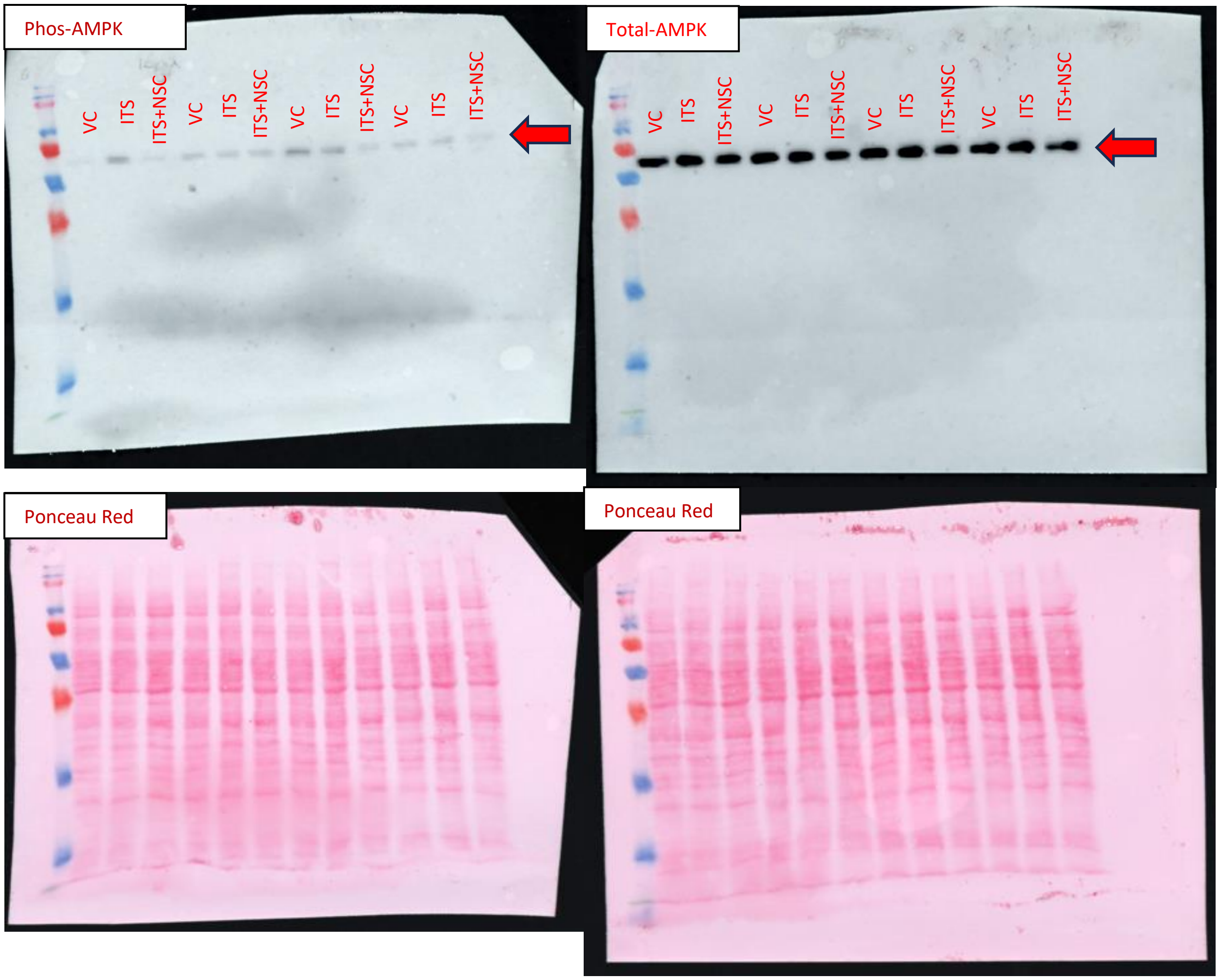

Supplemental Figure S5.F: Full images of phosphorylated AMPK (left) and total AMPK (right) in the ITS condition, without (ITS) or with (ITS+NSC) NSC185058 treatment. Full Ponceau S stains are presented below each image.

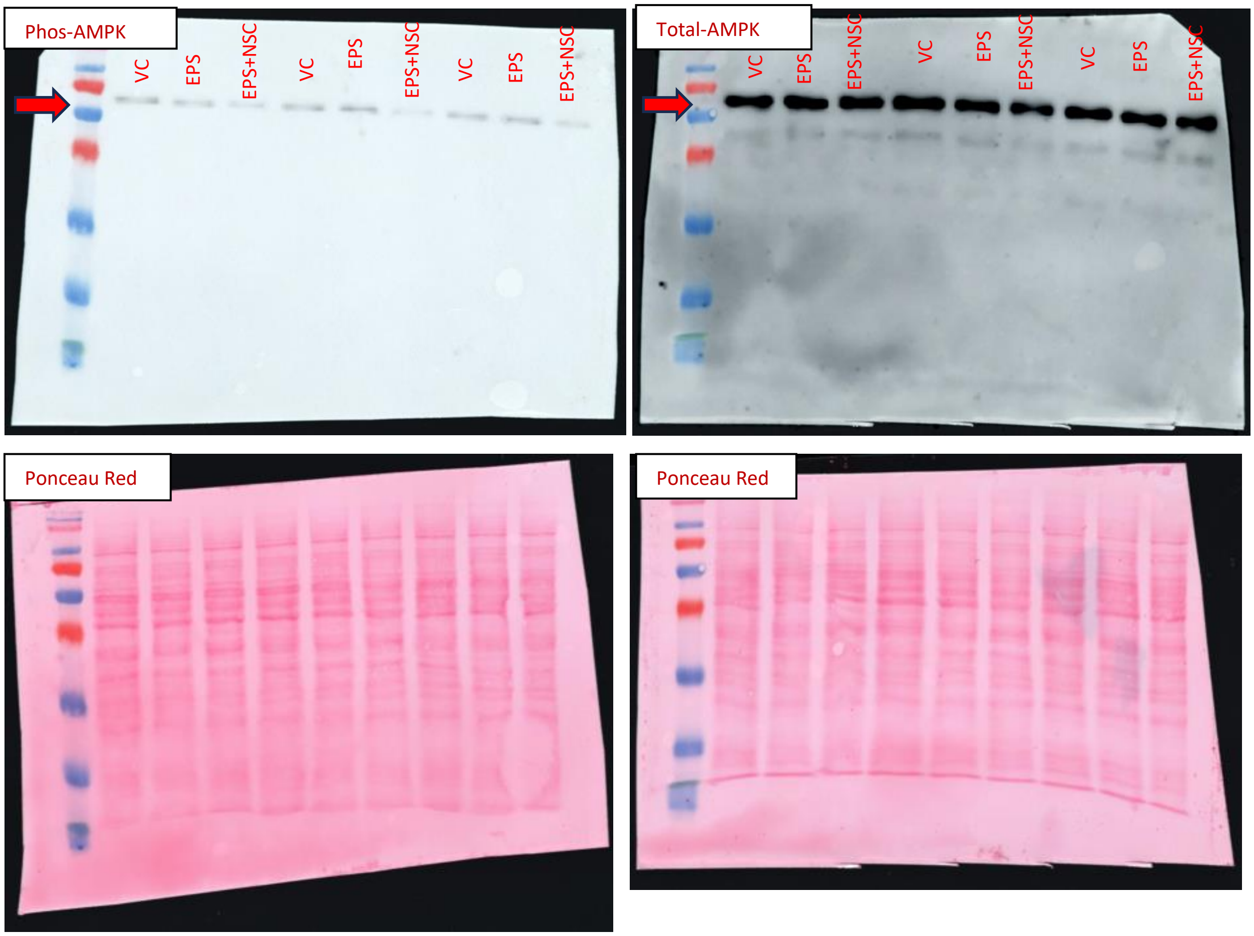

Supplemental Figure S5.G: Full images of phosphorylated AMPK (left) and total AMPK (right) in the EPS condition, without (EPS) or with (EPS+NSC) NSC185058 treatment. Full Ponceau S stains are presented below each image.

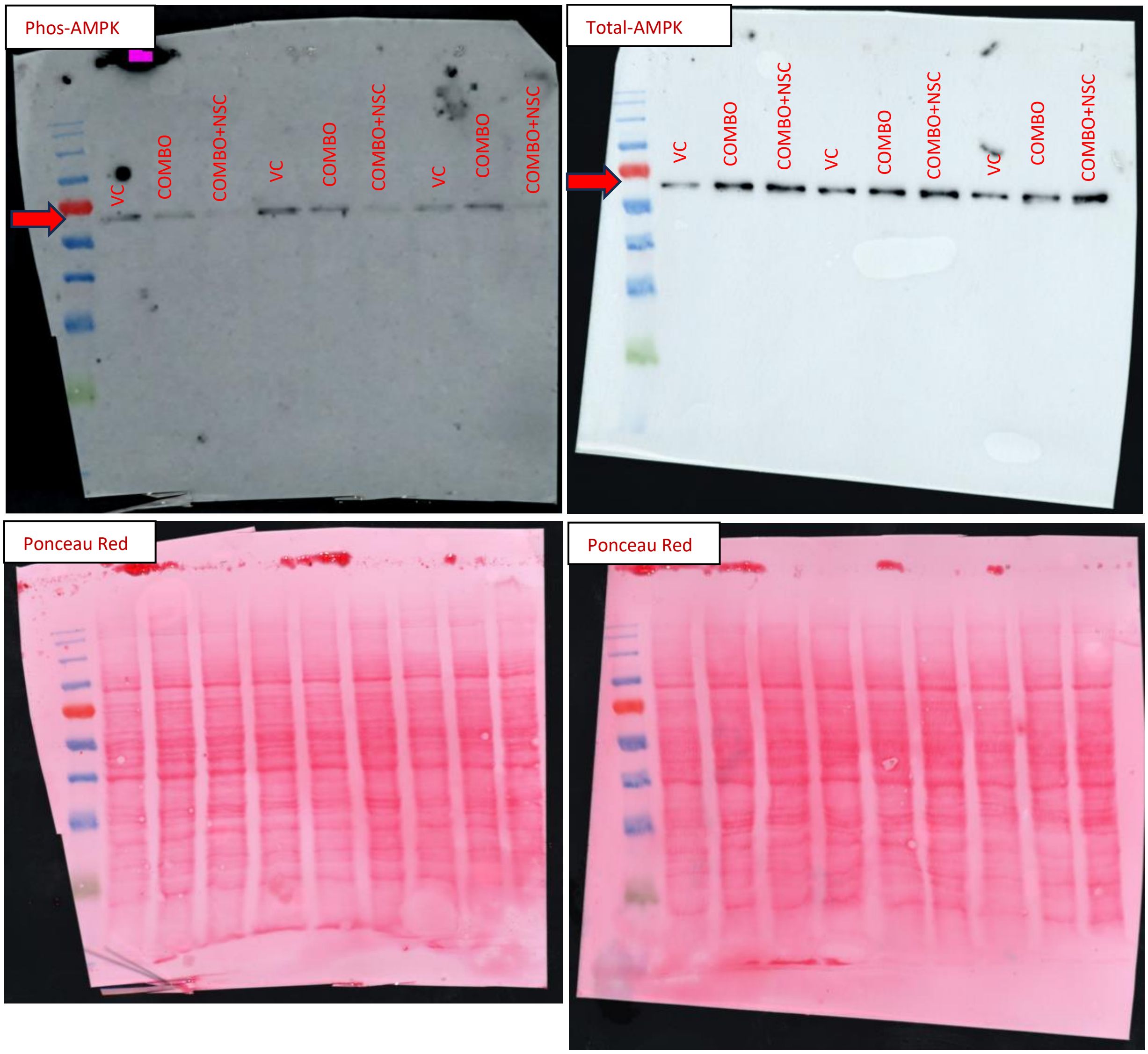

Supplemental Figure S5.H: Full images of phosphorylated AMPK (left) and total AMPK (right) in the Combo condition, without (Combo) or with (Combo+NSC) NSC185058 treatment. Full Ponceau S stains are presented below each image.

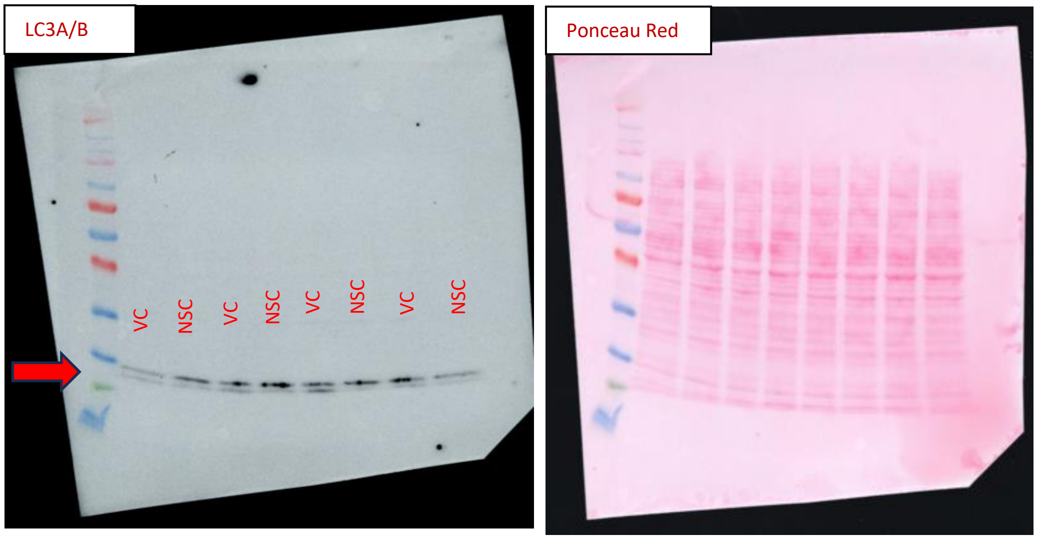

Supplemental Figure S6.A: Full image of LC3 in the Baseline condition, without (VC) or with (NSC) NSC185058 treatment. Full Ponceau S stain is presented to the right.

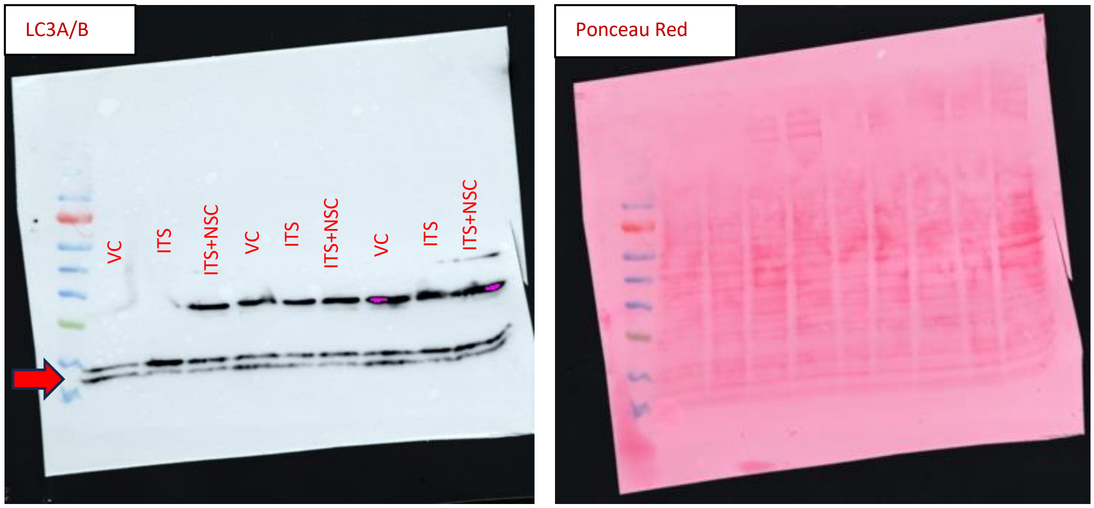

Supplemental Figure S6.B: Full image of LC3 in the ITS condition, without (ITS) or with (ITS+NSC) NSC185058 treatment. Full Ponceau S stain is presented to the right.

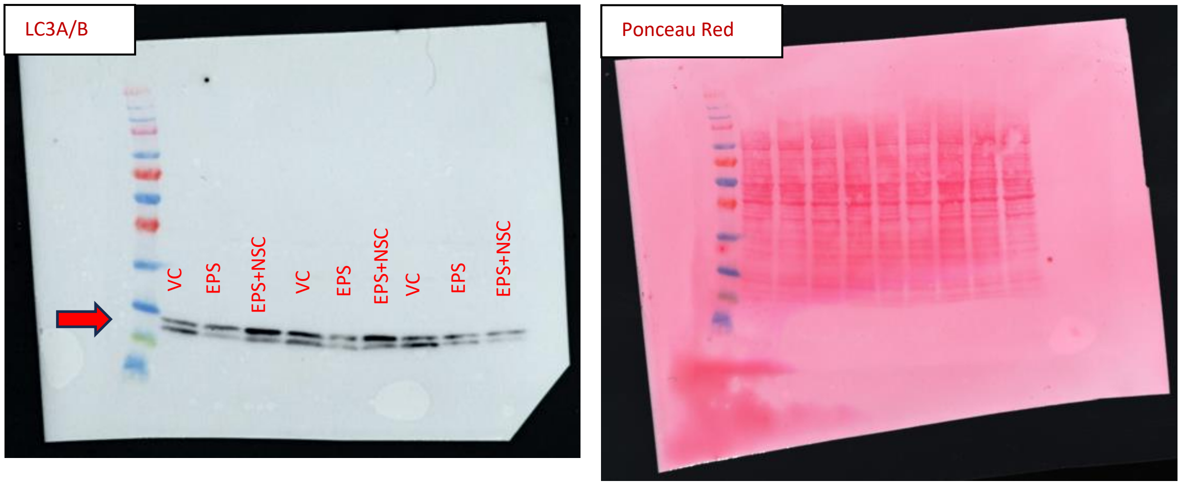

Supplemental Figure S6.C: Full image of LC3 in the EPS condition, without (EPS) or with (EPS+NSC) NSC185058 treatment. Full Ponceau S stain is presented to the right.

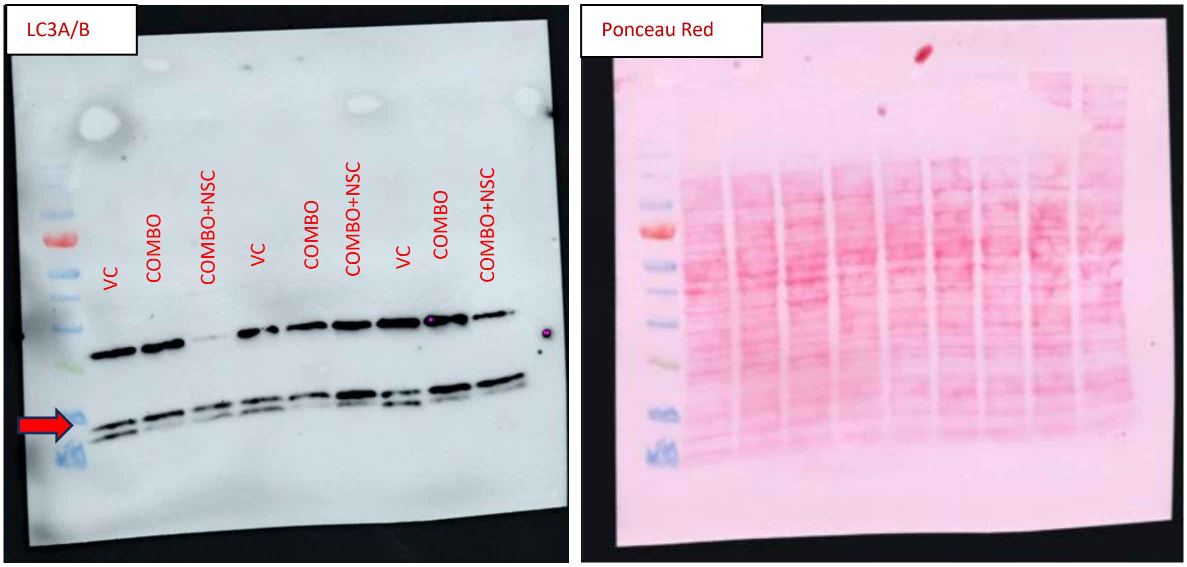

Supplemental Figure S6.D: Full image of LC3 in the Combo condition, without (COMBO) or with (COMBO+NSC) NSC185058 treatment. Full Ponceau S stain is presented to the right.

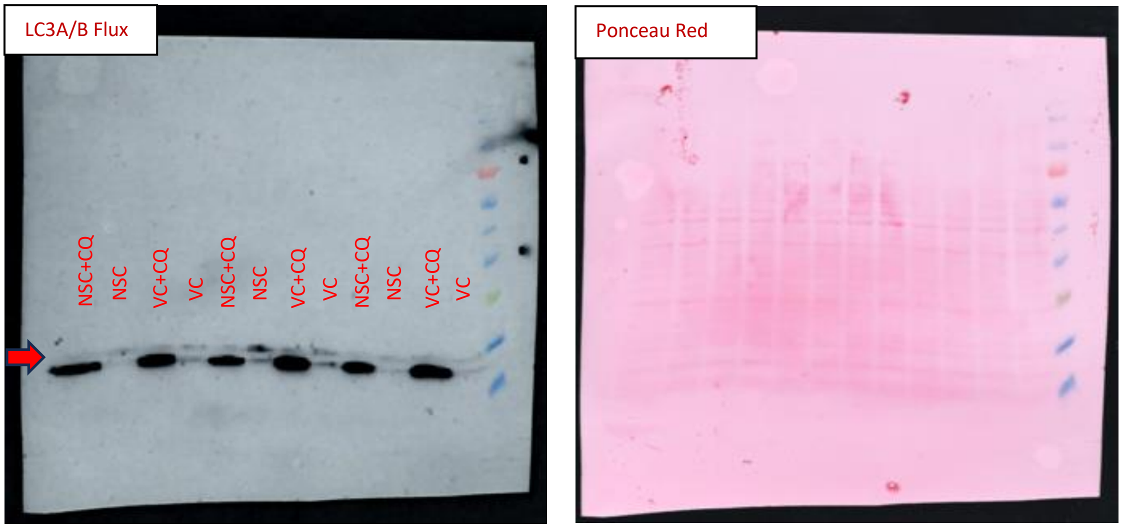

Supplemental Figure S6.I: Full image of LC3 flux in the Baseline condition, without (VC) or with (NSC) NSC185058 treatment, and without or with (VC+CQ, NSC+CQ) 40µM chloroquine to measure autophagic flux. Full Ponceau S stain is presented to the right.

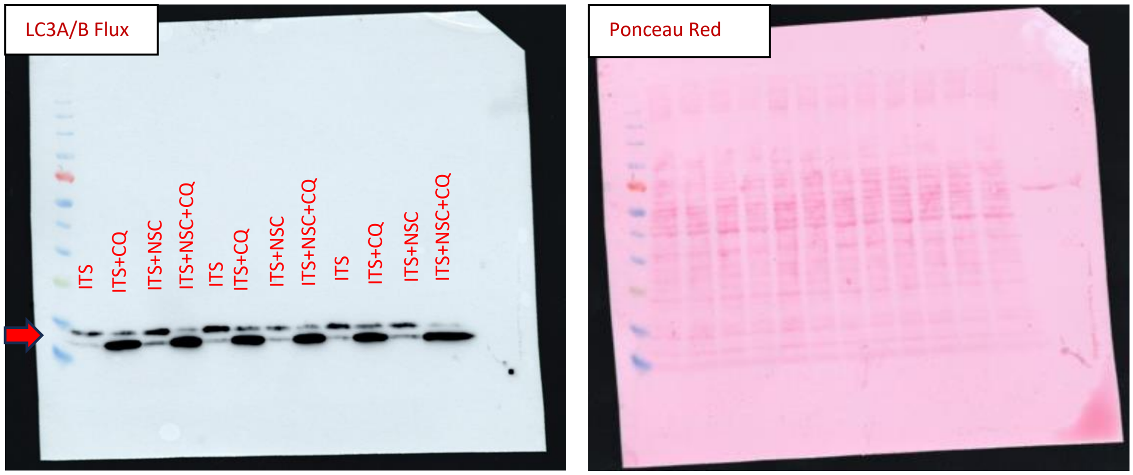

Supplemental Figure S6.J: Full image of LC3 flux in the ITS condition, without (ITS) or with (ITS+NSC) NSC185058 treatment, and without or with (ITS+CQ, ITS+NSC+CQ) 40µM chloroquine to measure autophagic flux. Full Ponceau S stain is presented to the right.

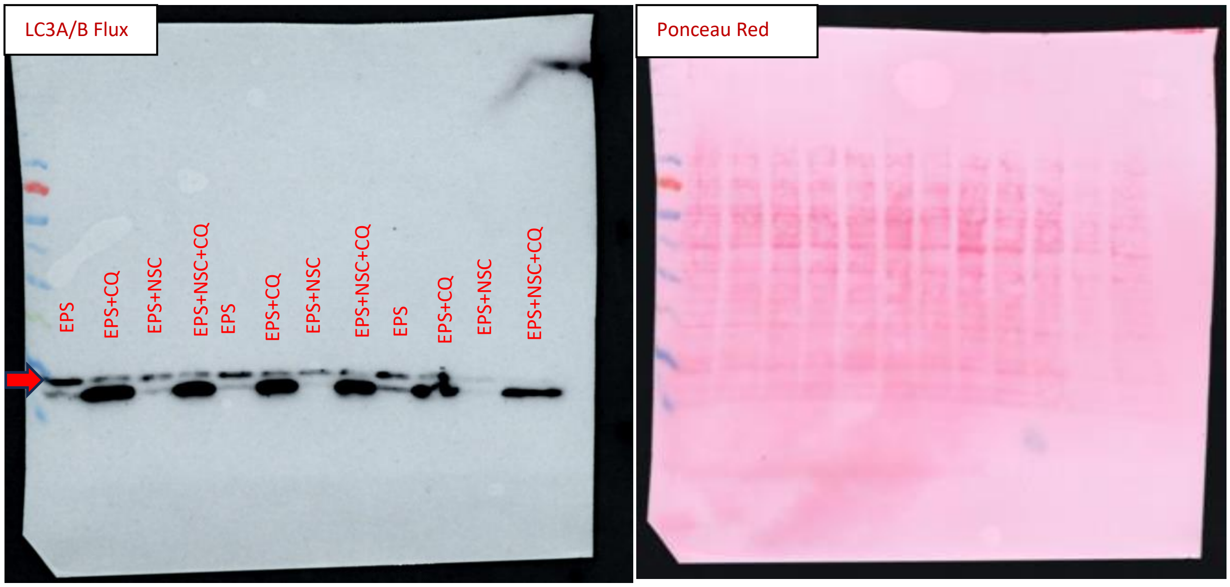

Supplemental Figure S6.K: Full image of LC3 flux in the EPS condition, without (EPS) or with (EPS+NSC) NSC185058 treatment, and without or with (EPS+CQ, EPS+NSC+CQ) 40µM chloroquine to measure autophagic flux. Full Ponceau S stain is presented to the right.

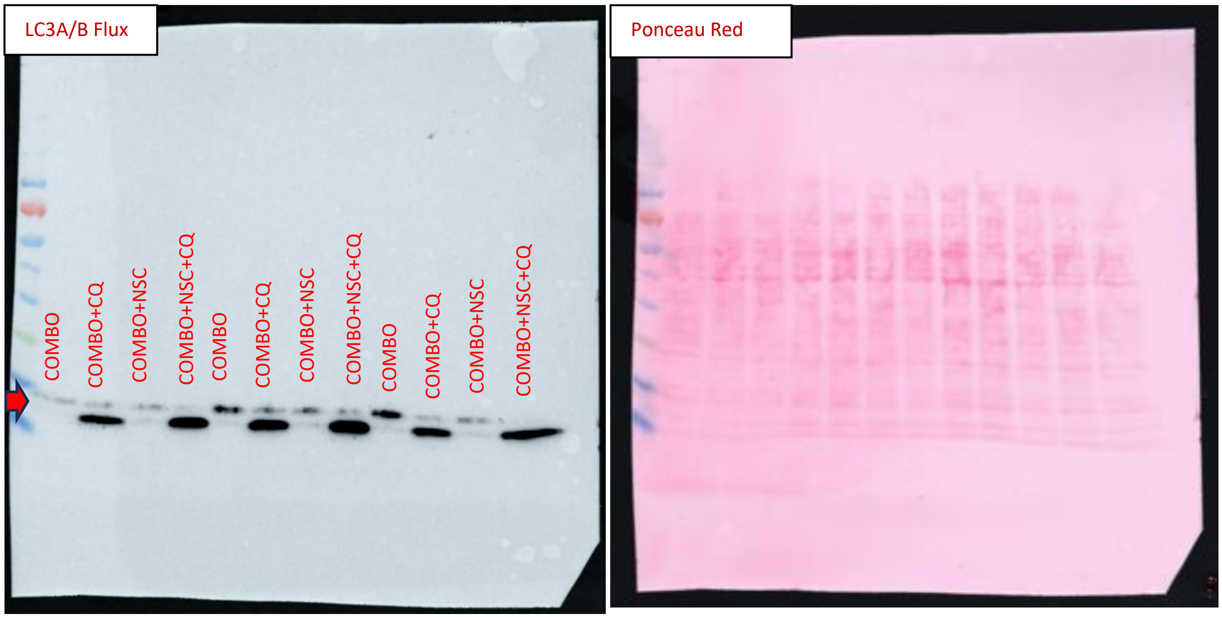

Supplemental Figure S6.L: Full image of LC3 flux in the EPS condition, without (COMBO) or with (COMBO+NSC) NSC185058 treatment, and without or with (COMBO+CQ, COMBO+NSC+CQ) 40µM chloroquine to measure autophagic flux. Full Ponceau S stain is presented to the right
